# Development of a senescence-related lncRNA signature in endometrial cancer based on multiple machine learning models

**DOI:** 10.3389/fgene.2025.1687922

**Published:** 2025-11-27

**Authors:** Jie Lin, Xuemei Lei, Yanhong Li, Xin Jiang, Fengle Jiang, Aihua Guo, Xintong Cai, Xingming Ye, Yang Sun

**Affiliations:** 1 Department of Gynecology, Clinical Oncology School of Fujian Medical University, Fujian Cancer Hospital, Fuzhou, Fujian Province, China; 2 Innovation Center for Cancer Research, Clinical Oncology School of Fujian Medical University, Fujian Cancer Hospital, Fuzhou, China; 3 Fujian Key Laboratory of Advanced Technology for Cancer Screening and Early Diagnosis, Fuzhou, China; 4 Department of Gynecology and Obstetrics, First Affiliated Hospital of Zhengzhou University, Zhengzhou, Henan, China; 5 Fujian Provincial Key Laboratory of Tumor Biotherapy, Clinical Oncology School of Fujian Medical University, Fujian Cancer Hospital, Fuzhou, Fujian Province, China

**Keywords:** endometrial cancer, cell senescence, signature, immunotherapy, prognosis, TCGA, patient-derived tumor organoids

## Abstract

**Background:**

Senescence-related lncRNAs (srlncRNA) mediate carcinogenesis in various malignancies. However, its roles in endometrial cancer (EC) remain unknown. Our research aims to construct a predictive srlncRNA model with prognostic and therapeutic significance in EC.

**Methods:**

We first downloaded the gene expression and medical information from the TCGA, as well as senescence-related lncRNAs (srlncRNAs) from the CellAge databases. Then, a co-expression network of cell senescence-related mRNA−lncRNA was explored with R. Subsequently, we performed Cox and Lasso regression and machine learning analysis to identify srlncRNAs related to the prognosis of EC and built a predictive model. Continually, we drew a nomogram to improve its ability to predict prognosis. Further, GSEA was used to explore potential mechanisms. Differences in TME, immune infiltrating cells, and checkpoints of the two risk groups were compared using GSEA and CIBERSORT. Finally, the drug sensitivity of patient-derived tumor organoids (PDOs) was investigated.

**Results:**

We first built a prognostic model based on seven srlncRNAs (AL121906.2, AP002761.4, BX322234.1, LINC00662, LINC00908, VIM-AS1, and ZNF236-DT). The model, which was screened by machine learning, functioned well in three sets with good stability and accuracy. Furthermore, the nomogram based on age, grade, and risk scores could precisely predict the prognosis of EC patients. The AUC of risk scores was highest compared to other clinical parameters (AUC risk score = 0.769, AUC age = 0.615, and AUC grade = 0.681). This srlncRNAs were enriched in the cell cycle, certain malignant tumors, and cancer-associated regulatory pathways. Afterward, low-risk EC patients had more immune-infiltrating cells and may benefit from anti-PD-1 and anti-CTLA4 treatment. Paclitaxel, gemcitabine, and cisplatin (all p < 0.05) may be more useful in EC patients with high expression of targeted srlncRNAs in the GDSC database. The levels of targeted srlncRNAs and drug sensitivity varied significantly among different EC PDOs. The EC-18 PDO was more resistant to three drugs, which aligned with clinical observation.

**Conclusion:**

The srlncRNA signature (AL121906.2, AP002761.4, BX322234.1, LINC00662, LINC00908, VIM-AS1, and ZNF236-DT) could guide prognosis prediction and treatment choices for EC patients.

## Introduction

Endometrial cancer (EC) is currently the most lethal gynecologic malignancy, with its incidence rising globally (Lin et al., 2024; Corr et al., 2025). According to the 2022 Global Cancer Observatory (GLOBOCAN), new cases of EC were estimated to reach 420,242, with mortality figures at 97,704 (Bray et al., 2024). It is projected that by 2050, more than 676,296 individuals worldwide will still be diagnosed with EC, resulting in nearly 183,083 fatalities (https://gco.iarc.who.int/tomorrow/en). The standard treatment for EC, according to NCCN guidelines, is a total hysterectomy along with bilateral salpingo-oophorectomy. Adjuvant therapies, such as radiation and/or chemotherapy, are considered based on the tumor’s histology and its stage (Abu-Rustum et al., 2025). Recently, molecular classification has brought immunotherapy, such as anti-PD-1, anti-PD-L1, or anti-CTLA-4 treatments, to the forefront (Abu-Rustum et al., 2025). While most EC patients present with early-stage disease and have favorable prognoses, patients with stage III and IV disease have 5-year survival rates of 48% and 15%, respectively (Crosbie et al., 2022). Specifically, approximately 20%–30% of ECs are considered high risk and are associated with recurrence rates of up to 30% at diagnosis (Gordhandas et al., 2023). Therefore, it is essential to focus on developing precise therapeutic strategies and effective prognostic biomarkers for patients with EC who are at high risk of recurrence.

Cellular senescence is a stress response that inhibits the growth of aged, damaged, or abnormal cells (McHugh et al., 2025). It can have both beneficial and detrimental effects in various physiological and pathological contexts, including cancer regulation (Schmitt et al., 2022; Chen et al., 2023; Deng et al., 2024; Zingoni et al., 2024; Colucci et al., 2025). A key molecular process involved in senescence is the progressive shortening of telomeres, the protective structures at the ends of chromosomes. This process ultimately leads to cellular senescence and can be influenced by senescence-related long non-coding RNAs (srlncRNAs) (Rossi and Gorospe, 2020). SrlncRNAs are typically over 200 nucleotides long. Although they do not code for proteins, they play important roles in immune remodeling and cancer regulation across various types of cancer (Santos-Sousa et al., 2025), including melanoma (Lin et al., 2023), head and neck squamous cell carcinoma (Chen et al., 2024), and glioma (Liu et al., 2023). To date, there has been limited research focused on the roles of srlncRNAs in EC.

To fill this gap, we construct a predictive srlncRNAs RNA signature based on the Cancer Genome Atlas (TCGA) database and the CellAge database by using machine learning analysis. Then, we investigate its predictive abilities in prognosis, potential biological functions and mechanisms, and immune status. Finally, the drug sensitivity of patient-derived tumor organoids (PDOs) was investigated as external validation.

## Materials and methods

### Data source

Gene expression and clinical medical information for EC patients were retrieved from the TCGA-UCEC database (https://cancergenome.nih.gov/). The raw data were normalized using R (version 3.6.3). Initially, 552 patients were selected for analysis. However, after excluding samples with incomplete or duplicated clinical features—such as age, stage, TNM classification, and overall survival—a total of 503 samples were included in our study. Detailed clinical features can be found in Supplementary Table S1. Additionally, we extracted 279 senescence-related mRNAs from the CellAge database (https://genomics.senescence.info/cells/cellAge.zip) for further analysis. The data were downloaded on 1 May 2022.

### Construction of predictive signature

A senescence-related lncRNA-mRNA co-expression gene expression network was established with absolute Pearson correlation coefficient values |Coef| of >0.3 and *P* < 0.001 as the thresholds. To visualize the lncRNA-mRNA co-expression network, a Sankey diagram was created using the R package (version 3.6.3). A total of 974 senescence-related lncRNAs were identified. After filtering out srlncRNAs related to the prognosis, we created a prognostic model by performing Kaplan–Meier and univariate and multivariate Cox regression analyses and Lasso analysis using machine learning analysis. Finally, a receiver operating characteristic curve (ROC) was drawn to evaluate the model’s predictive ability. We also identified whether the signature was an independent risk factor by Cox analyses.

The risk score was calculated by the following formula (Lin et al., 2025): Risk score = ∑i β_1_ (lncRNA_1_) × EXP (lncRNA_1_) + β2 (lncRNA_2_) × EXP (lncRNA_2_) +……+ β n (lncRNA_n_) × EXP (lncRNA_n_), EXP represents the expression level of srIncRNAs in our model, β represents the coefficient of srIncRNAs in the multivariate Cox regression analysis. According to the median risk score, EC patients were divided into the high- and low-risk groups.

### Signature validation and nomogram assessment

We developed an optimal predictive model using machine learning techniques, including GBM (Gradient Boosting Machine), Random Survival Forest (RSF), CoxBoost, SurvivalSVM, and Lasso regression. We randomly divided 503 EC patients into a training set (n = 251) and a test set (n = 252) to evaluate the model’s reliability and accuracy. We compared the risk score distributions, ROC curves, and overall survival (OS) between the two groups across three sets. Additionally, we created a nomogram that incorporates risk scores, age, and tumor grade. The calibration curves for 1-, 3-, and 5-year survival demonstrated the predictive capability of the nomogram.

### GSEA analysis and immune status investigation

We conducted Gene Ontology (GO) (Nucleic Acids Res 43, 2015) and Kyoto Encyclopedia of Genes and Genomes (KEGG) analyses (Kanehisa and Goto, 2000), as well as GSEA (https://www.broadinstitute.org/gsea/index.jsp) to explore the potential mechanisms and pathways between the two groups. To evaluate the immunotherapy response of the two risk groups, we analyzed and compared the differences in the tumor microenvironment (TME), immune cells, and immune checkpoints. The TME (stromal, immune, and ESTIMATE scores) of each sample in the two risk groups was assessed using ESTIMATE (Yoshihara et al., 2013). We then used a single-sample gene set enrichment analysis (ssGSEA) (Hänzelmann et al., 2013) and the CIBERSORT(Newman et al., 2019) algorithm to investigate the tumor-infiltrating immune cells between the two groups. Moreover, the immune checkpoints between groups were also compared.

### PDOs culture

The fresh tumor tissues were obtained from EC patients who were undergoing surgical treatment. Their clinical information is provided in Supplementary Table S2. The procedure for PDO culture is described in our previous experiments (Cai et al., 2023). Fresh tumor tissue was cut into 1 mm^3^ and transferred to a 6 cm dish containing 4 mL Tumor Tissue Digestion Solution (Mogengel Bio; Cat: MB-0818L05). The mixture was digested on a shaker at 80 rpm at 37 °C for 30 min to 1 h until no granular tissue was evident. Digestion was stopped by the addition of DPBS. The solution was filtered through a 100 μm cell filter to remove debris, then centrifuged at 300 *g* for 3 min at 4 °C, and the supernatant was discarded. Pellets were resuspended in Human Endometrial Cancer Organoid Kit (Mogengel bio; Cat: MA-0807T007), then mixed with 1:1 Matrigel (Mogengel bio; Cat:082703), and 50 μL of each drop was inoculated into preheated 6-well plates. Incubate at 37 °Cand 5% CO_2_ for 15–30 min to polymerize the matrix. After polymerization, 2 mL of organoid medium was added to each well for continuous culture. The morphology of PDOs was observed every 2 days by the microscope (Nikon, TS2). Organoids were dissociated at 37 °C for 5 min when they grew to 150–200 µm using organoid dissociation solution TrypLE™ Express (Gibco; Cat:12605028). Then we passed them to the first (P1) and second generations (P2).

### RT-qPCR validation

We extracted total RNA from the PDOs following the instructions provided with the RNAeasy™ Animal RNA Extraction Kit (Beyotime; Cat: R0026). cDNA was synthesized through reverse transcription using the NovoScript® Plus All-in-one 1st Strand cDNA Synthesis SuperMix (gDNA Purge) (Novoprotein; Cat: E047). GAPDH served as the internal reference. The amplification process comprised 40 cycles, starting with an initial denaturation step at 95 °C for 5 minutes. This was followed by 10 s at 95 °C and then 30 s at 60 °C. The mRNA expression levels were normalized to GAPDH using the ChamQ Blue Universal SYBR qPCR Master Mix (Vazyme; Cat: Q312) (Ji et al., 2023). The primers utilized are listed in Supplementary Table S3.

### Immunohistochemistry (IHC) validation

The IHC of tumor tissues and organoids was performed to validate their origin consistency with specific biomarkers associated with EC, including Estrogen Receptor (ER), Progesterone Receptor (PR), and Vimentin. Additionally, Ki-67 was assessed to identify tumor malignant behaviors. The antibodies used in this study are listed below: ER (Proteintech; Cat: 21244-1-AP; 1:500), PR (Proteintech; Cat: 25871-1-AP; 1:500), Vimentin (Servicebio; Cat: GB121308; 1:1000), and Ki-67 (Servicebio; Cat: GB111499; 1:1000).

Immunohistochemical staining for each slide was evaluated blindly by two pathologists using a semi-quantitative method. For each sample, three random fields of view at ×100 magnification were selected for scoring. Staining intensity was scored based on the degree of positive staining: intense (3 points), moderate (2 points), weak (1 point), and negative (0 points). The proportion of stained cells was categorized into four groups: 76%–100% (4 points), 51%–75% (3 points), 26%–50% (2 points), and 0%–25% (1 point). The immunohistochemical H-score for each sample was calculated by multiplying the intensity score by the percentage of positively stained cells.

Samples were classified into four groups based on their immunoreactive scores: 0 (negative, −), 1–4 (weakly positive, +), 5–8 (positive, ++), and 9–12 (strongly positive, +++). The concordance of immunohistochemical staining between tumor tissue and PDOs was assessed using the Pearson correlation coefficient (r = 0.8651, *p* < 0.05). The agreement between the two pathologists’ assessments was evaluated using the Kappa test (κ = 0.869, *p* < 0.05), which indicates good agreement.

### Drug sensitivity in PDOs

First, we retrieved drug information, including paclitaxel, gemcitabine, and cisplatin, from the Genomics of Drug Sensitivity in Cancer (GDSC) database (https://www.cancerrxgene.org). Half maximal inhibitory concentration (IC_50_) was used to predetermine drug sensitivity for EC patients. Then, the PDOs from different EC patients were cultured and exposed to various concentrations of Paclitaxel (GLPBIO; Cat: GC12511), Gemcitabine (GLPBIO; Cat: GC14447), and Cisplatin (MCE; Cat: HY-17394) at concentrations of 100, 10, 1, 0.1, and 0.01 μg/mL for 120 h, respectively. The 0.1% DMSO was used as the control group. The drug sensitivity of organoids was measured by ATP using CellTiter-Lumi™ Luminescence 3D Cell Viability Detection Kit (Beyotime; Cat: C0061).

### Statistical analysis

R (version 3.6.3) was utilized for data analysis. The Kaplan-Meier plots and Cox regression analysis were conducted using the “survival” and “survminer” packages. GBM machine learning analysis was performed using the “GBM” package, while RSF machine learning analysis was conducted using the “randomForestSRC” package. The CoxBoost machine learning analysis utilized the “CoxBoost” package, and the SurvivalSVM analysis was conducted using the “survivalsvm” package. GraphPad Prism (version 10.1.2) was employed for statistical analyses of the experiments. All experiments were performed in triplicate, and a significance level of *p* < 0.05 was considered statistically significant.

## Results

### The construction and validation of srlncRNA signature

**FIGURE 1:**
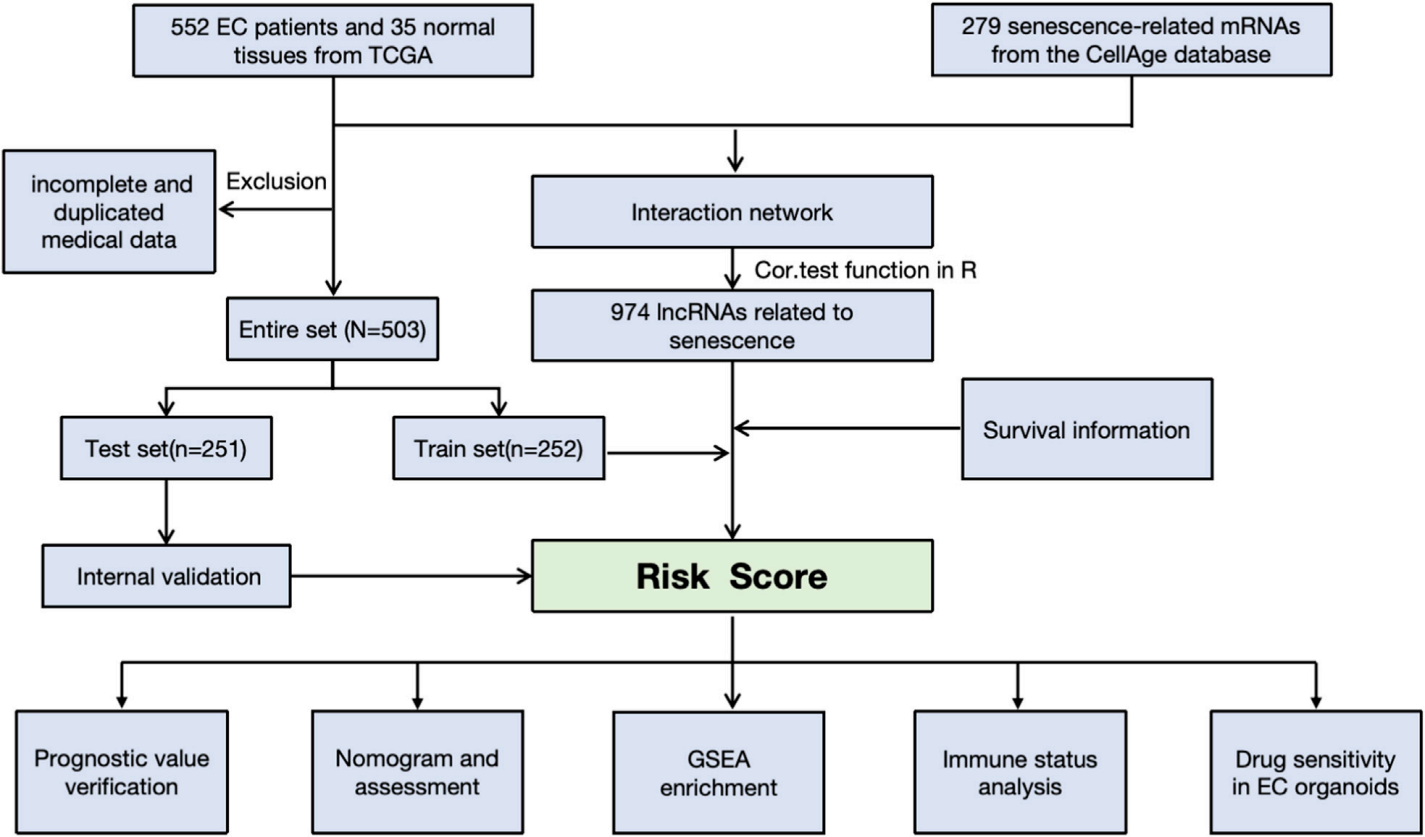
Flow diagram of the study.

**FIGURE 2:**
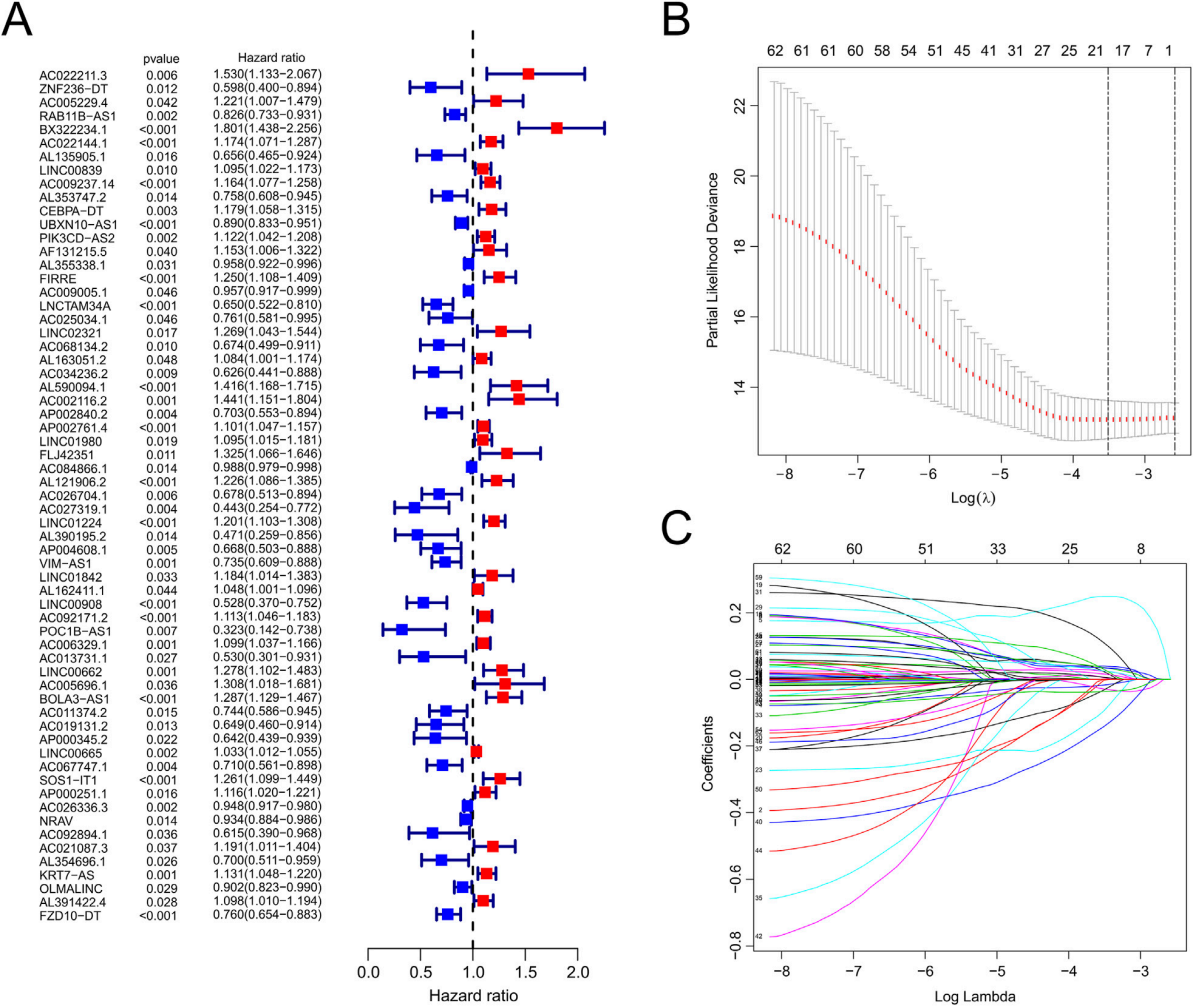
Identification of srlncRNAs with significant prognostic value in EC. **(A)** The forest showed the HR (95%CI) and p-value of selected lncRNAs by univariate Cox proportional-hazards analysis. **(B,C)** Lasso regression.

**FIGURE 3:**
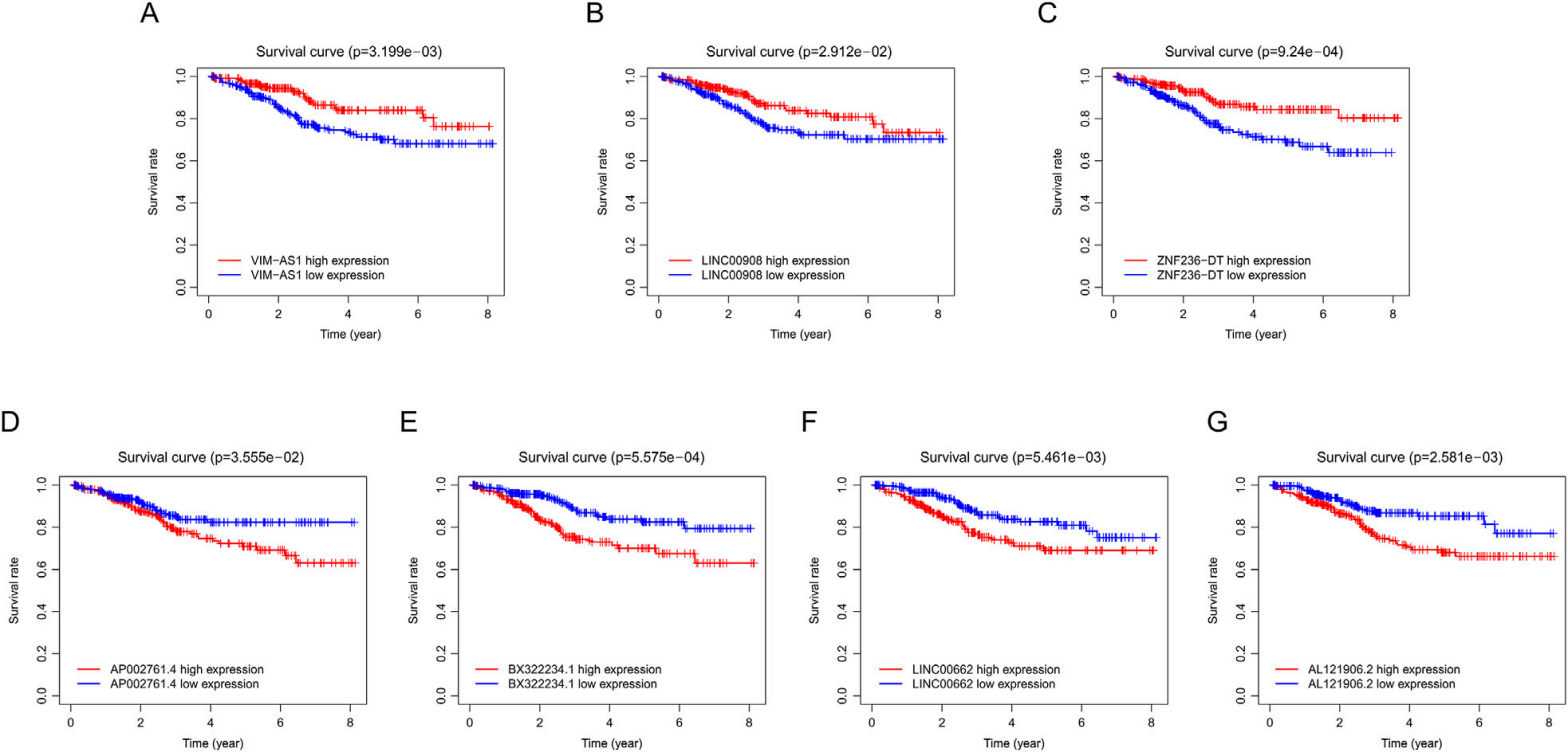
Survival curve of EC patients in different groups. **(A)** Comparison of survival rates between high and low VIM-ASI levels in EC patients using Kaplan-Meier analysis. **(B)** Comparison of survival rates between high and low LINC00908 levels in EC patients using Kaplan-Meier analysis. **(C)** Comparison of survival rates between high and low ZNF236-DT levels in EC patients using Kaplan-Meier analysis. **(D)** Comparison of survival rates between high and low AP002761.4 levels in EC patients using Kaplan-Meier analysis. **(E)** Comparison of survival rates between high and low BX322234.1 levels in EC patients using Kaplan-Meier analysis. **(F)** Comparison of survival rates between high and low LINC00662 levels in EC patients using Kaplan-Meier analysis. **(G)** Comparison of survival rates between high and low AL121906.2 levels in EC patients using Kaplan-Meier analysis. EC, endometrial cancer.

**FIGURE 4:**
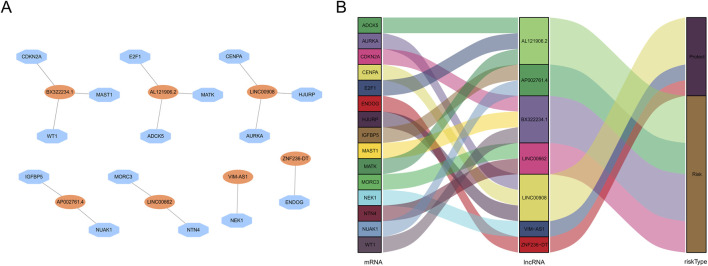
Screening of prognostic srlncRNA in EC. **(A)** A co-expression network of srlncRNAs and mRNAs. **(B)** The Sankey diagram of the relationship between lncRNA and mRNA.

The workflow is demonstrated in Figure 1. Nine hundred forty-seven srlncRNAs were initially identified. Sixty-three out of these identified srlncRNAs were significantly associated with the prognosis of EC (*p* < 0.01). Among sixty-three srlncRNAs, thirty-one were linked to low risk (hazard ratio (HR) < 1), and thirty-two were related to high risk (HR > 1). Subsequently, we anchored seven srlncRNAs to create a predictive model by performing Lasso regression and Cox regression analysis (Figure 2). Those genes were LINC00908, VIM-AS1, and ZNF236-DT, AL121906.2, AP002761.4, BX322234.1, and LINC00662. Among these seven srlncRNAs, three srlncRNAs (LINC00908, VIM-AS1, and ZNF236-DT) were beneficial for prognosis, whereas the other four srlncRNAs (AL121906.2, AP002761.4, BX322234.1, and LINC00662) were detrimental to prognosis (Figure 3; Supplementary Table S4). By machine learning, the Lasso model with seven srlncRNAs was identified to be optimal (Supplementary Table S5). A prognostic visual co-expression network of srlncRNAs–mRNAs was exhibited (Figure 4).

**FIGURE 5:**
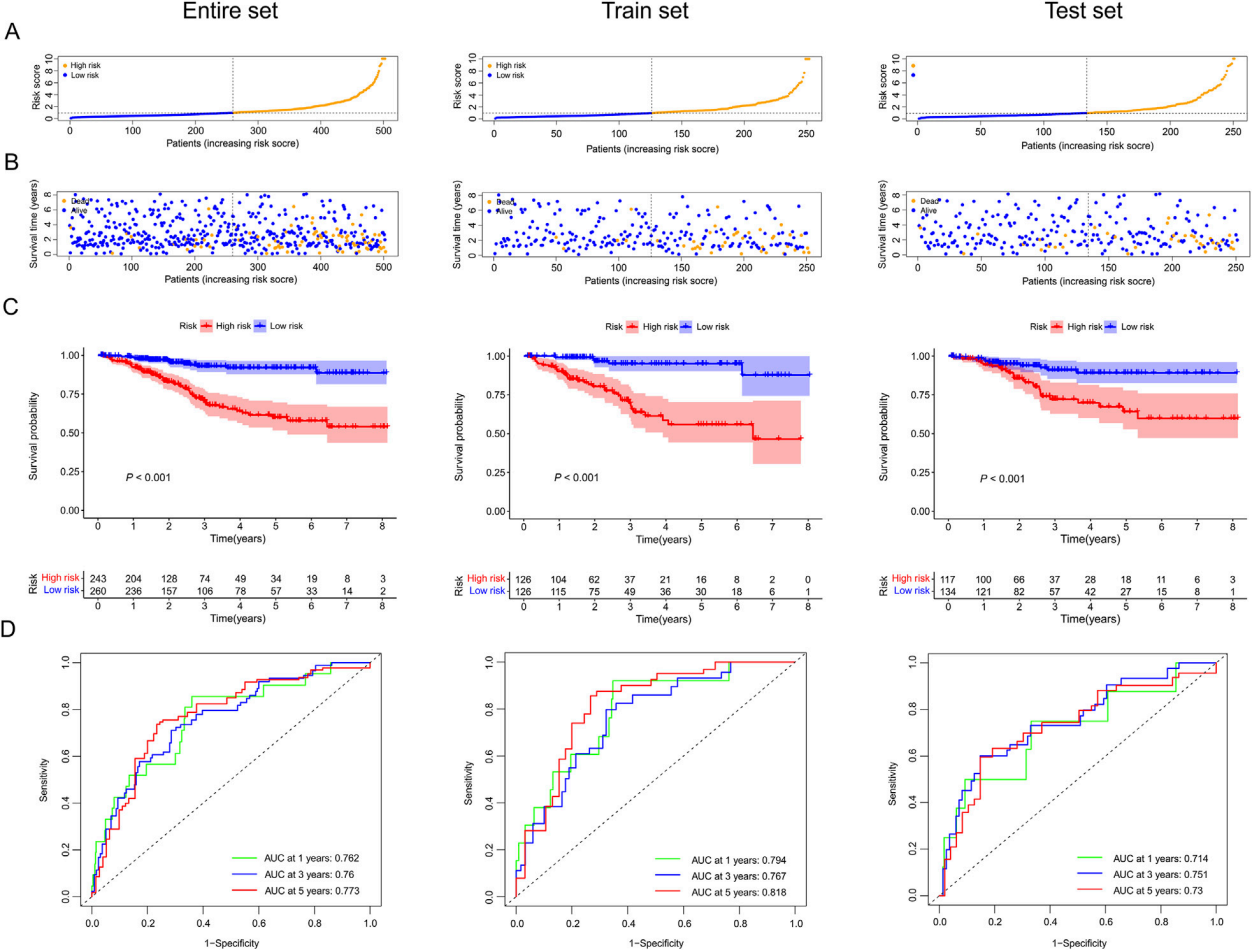
The prognostic value of our predictive model in the entire, train, and test sets. **(A)** Exhibition of risk scores of the low and high-risk groups in three sets. **(B)** Survival time and survival status of EC patients in low and high-risk groups of three sets. **(C)** Kaplan-Meier survival curves for EC patients in low- and high-risk groups across three sets. **(D)** Time-dependent ROC curves between low and high-risk groups in three sets.

We contrasted the distribution of risk scores, the survival status, the ROC curve, and the survival outcomes in three sets, respectively (Figure 5; Supplementary Figure S1). The risk curves and scatter plots implied that mortality was positively associated with the risk score in three sets (Figures 5A,B). We also found that the patients in the high-risk group had lower OS rates than patients with lower risk scores in three sets (all *p* < 0.05, Figure 5C). Moreover, ROC curves of the entire set (ROC_1-year_ = 0.762, ROC_3-year_ = 0.76, ROC_5-year_ = 0.773), train set (ROC_1-year_ = 0.762, ROC_3-year_ = 0.76, ROC_5-year_ = 0.773), and test set (ROC_1-year_ = 0.714, ROC_3-year_ = 0.751, ROC_5-year_ = 0.730) all demonstrated our prognostic model was stable and reliable (Figure 5D).

### Prognosis value and nomogram assessment

**FIGURE 6:**
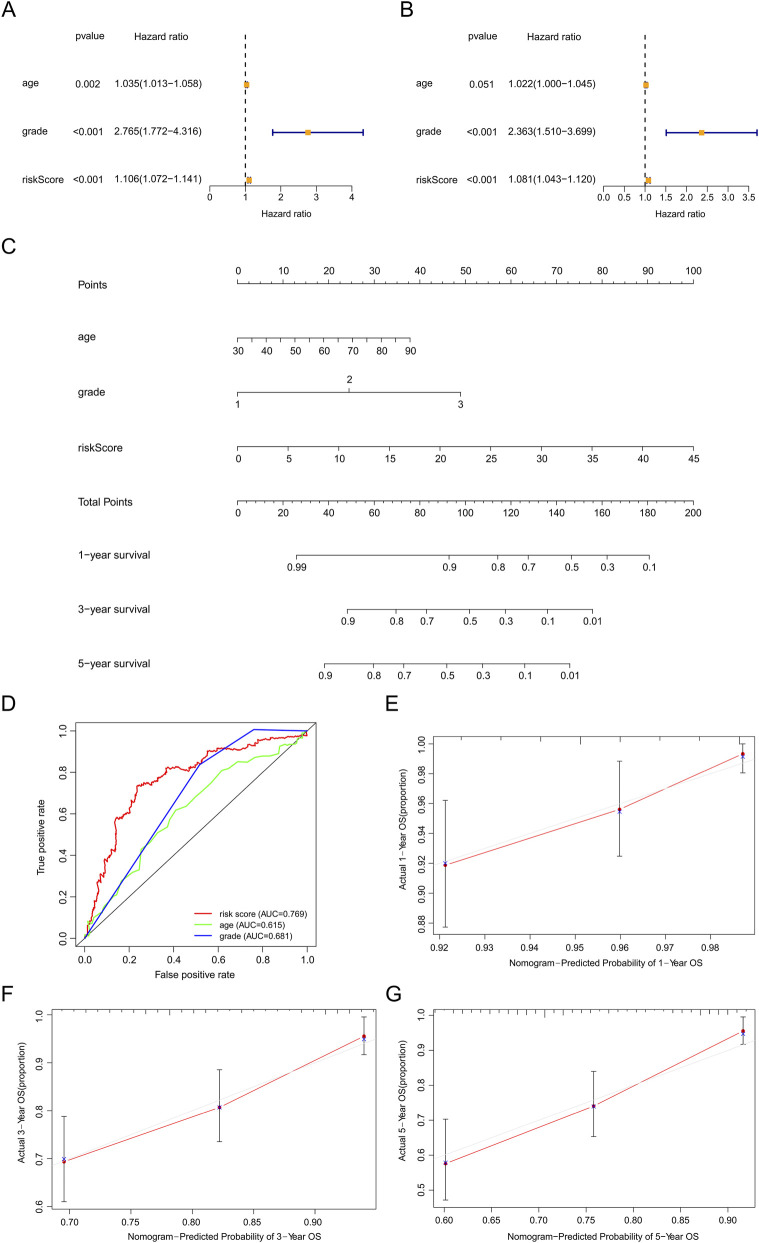
Assessment of the prognostic survival model based on seven srlncRNAs. **(A,B)** Univariate and Multivariate Cox regression analysis of risk score and clinical factors. **(C)** The nomogram of risk score and clinical factors. Clinical factors: age and grade. **(D)** The AUC for risk model score, age, and grade. Clinical factors: age and grade. **(E)** The 1-year OS calibration curve. **(F)** The 3-year OS calibration curve. **(G)** The 5-year OS calibration curve.

Based on the results of univariate and multivariate Cox regression analyses, we found the HR of risk score was 1.106 (Figure 6A) and 1.081 (Figure 6B), respectively (all *p* values <0.001). These results indicated that our predictive model was an independent prognostic factor for EC patients. The AUC value was 0.769, higher than age and grade (Figure 6D), suggesting that our model could more precisely predict the survival outcomes of EC patients. Afterward, the nomogram we created was easy to master and could precisely predict the 1-, 3-, and 5-year survival probability (Figure 6C). Calibration curves validated our model’s predictive ability (Figures 6E–G).

**FIGURE 7:**
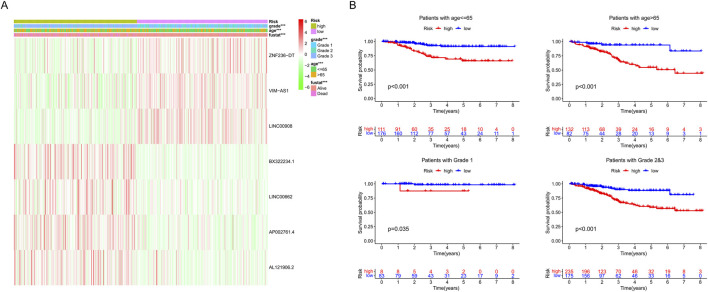
**(A)** Heat map of seven srlncRNAs’ expression. **(B)** Survival analysis of the high-risk and low-risk groups in patients under different subgroups (age ≤ 65, age >65, grades 1, and grades 2–3).

We examined the correlation between model genes, risk score, age, fustat, and grade. High levels of three favorable lncRNAs (ZNF236-DT, VIM-AS1, and LINC00908) were negatively correlated with risk scores, grade, age, and mortality rates. In contrast, high levels of the other four harmful lncRNAs (BX322234.1, LINC00662, AP002761.4, and AL121906.2) were positively correlated with them (Figure 7A). Furthermore, we found that patients in the high-risk group had lower OS, compared to those in the low-risk group, based on different subgroups (age ≤65 years or >65 years and grade 1 or grades 2–3) (Figure 7B). These results indicated that the risk score was efficient in predicting the prognosis of EC patients.

We investigated the prognostic value and immune response of the srlncRNA signature across different TCGA molecular subtypes. Our findings indicate that EC patients with the CNH subtype have a higher risk score. In contrast, patients with the POLE, MSI, and CNL subtypes show lower risk scores, reflecting a similar trend in prognostic prediction and immune response with TCGA molecular subtypes. Specifically, patients with high-risk scores tend to have worse survival outcomes compared to those with low-risk scores, particularly in the MSI subtype (*p* < 0.0001) and the POLE subtype (*p* = 0.035). Therefore, our scoring system may be valuable for predicting prognostic significance and immune response when combined with TCGA subtypes (Supplementary Table S6; Supplementary Figure S2).

### GSEA analysis

**FIGURE 8:**
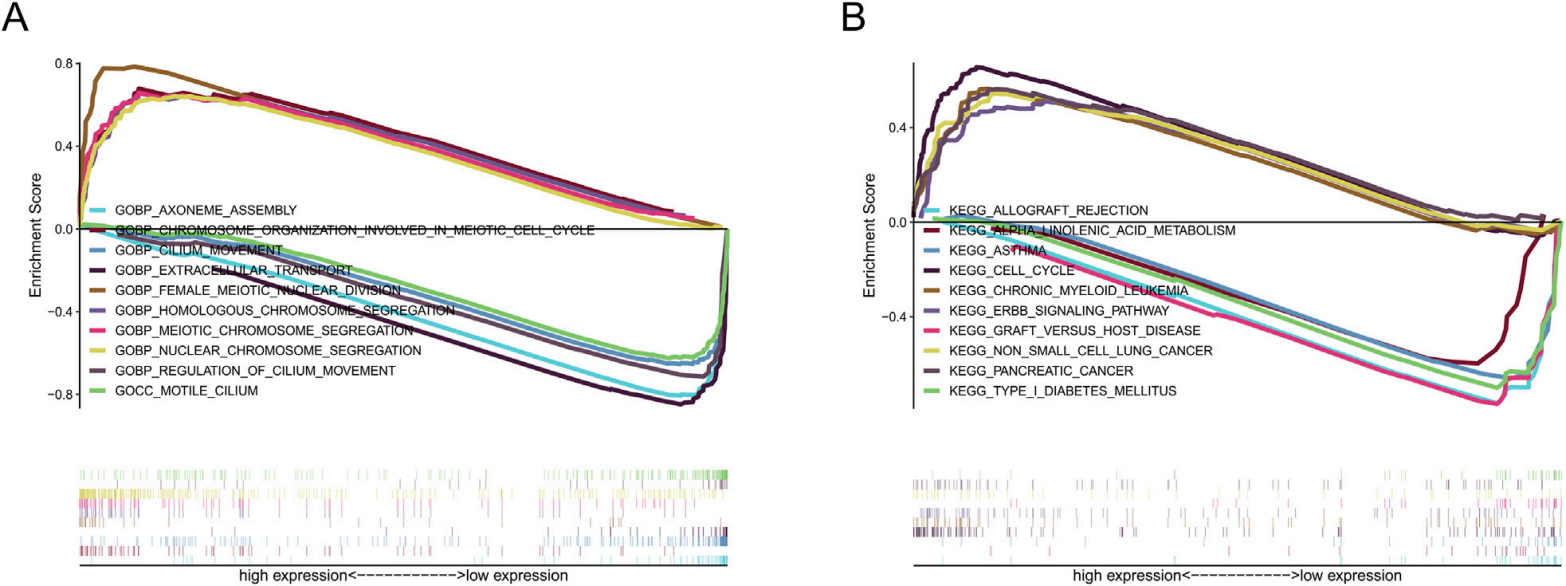
The results of functional analysis based on seven srlncRNAs model by GSEA. **(A)** GO enrichment analysis. **(B)** KEGG enrichment analysis.

GO analysis revealed that nuclear chromosome segregation and extracellular transport were the primary biological functions of our identified srlncRNAs (Figure 8A). At the same time, KEGG analysis revealed srlncRNAs with high-risk scores were enriched in cell cycle, tumor regulations, and classical tumor-related pathways, such as the Erbb signaling pathway. In contrast, srlncRNAs with low-risk scores were concentrated in immune rejection (Figure 8B).

### Immune landscape investigation

**FIGURE 9:**
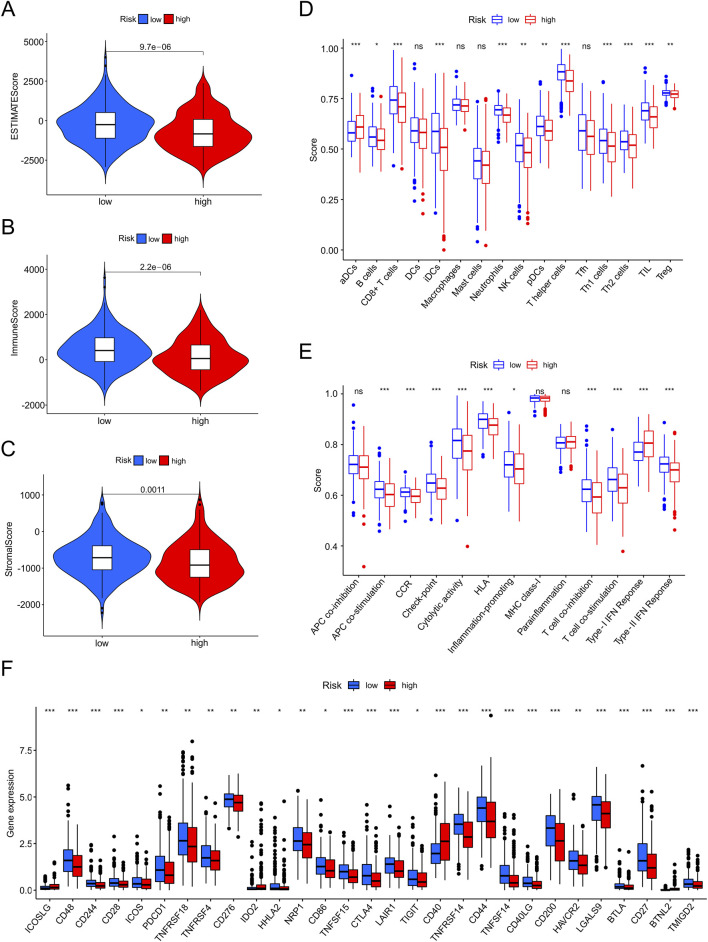
The investigation of tumor immune factors and immunotherapy. **(A–C)** Estimate score, immune score, and stromal score in high-and low-risk groups. **(D,E)** The varied proportions of immune cells and immune functions in high-and low-risk groups by ssGSEA. **(F)** The 29 immune checkpoint inhibitor (ICI) levels in high-and low-risk groups.

The tumor immune microenvironment of EC was investigated using ESTIMATE. The TME scores (ESTIMATE, immune, and stromal scores) were lower in the high-risk group than in the low-risk group (all *p* < 0.050), indicating that the immune system was more vigorous in patients with lower-risk scores (Figures 9A–C). Furthermore, we used ssGSEA to assess immune-infiltrating cells and immune functions between the two risk groups (Figures 9D,E). The abundance of B cells (*p* < 0.05), CD8^+^ T cells (*p* < 0.001), iDCs (*p* < 0.001), neutrophils (*p* < 0.001), NK cells (*p* < 0.01), pDCs (*p* < 0.01), T helper cells (*p* < 0.01), Th1 cells (*p* < 0.001), Th2 cells (*p* < 0.001), TILs (*p* < 0.001) and Tregs (*p* < 0.01) were observed in low-risk patients. In contrast, the aDCs were higher in patients with high-risk scores (*p* < 0.001). In the low-risk group, a total of 25 checkpoints were upregulated, such as CD28, CD86, CTLA4, TIGIT, CD44, and CD27 (*p* < 0.05), whereas in the high-risk group, four therapy sites (ICOSLG, IDO2, CD40, and BTNL2) were upregulated (*p* < 0.05) (Figure 9F).

### Drug sensitivity in PDOs

**FIGURE 10:**
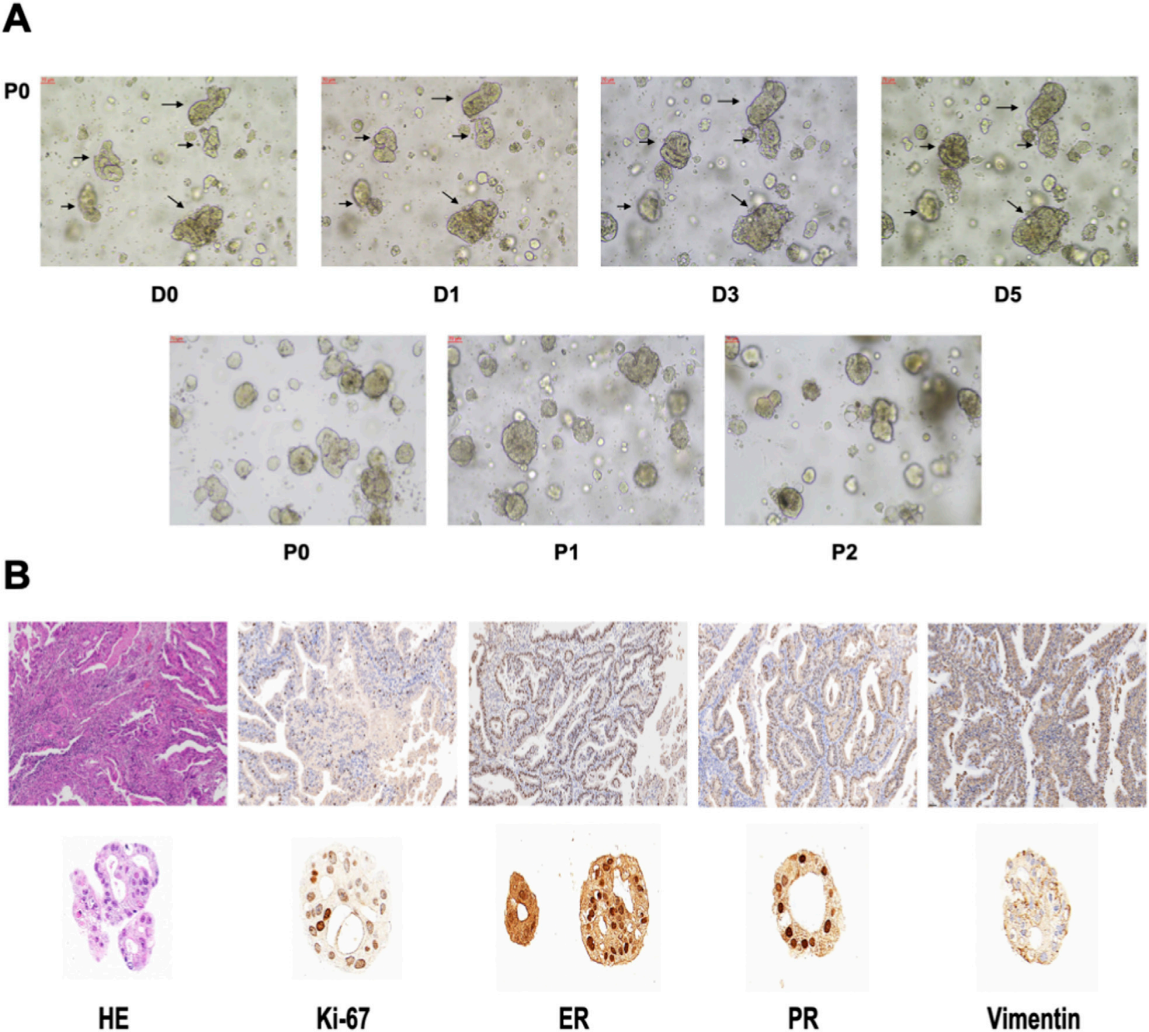
**(A)** The organoid morphology of P0, P1, and P2. **(B)** Comparison of HE and IHC between organoids and EC tissues. HE, Hematoxylin-eosin staining. IHC, Immunohistochemistry.

**FIGURE 11:**
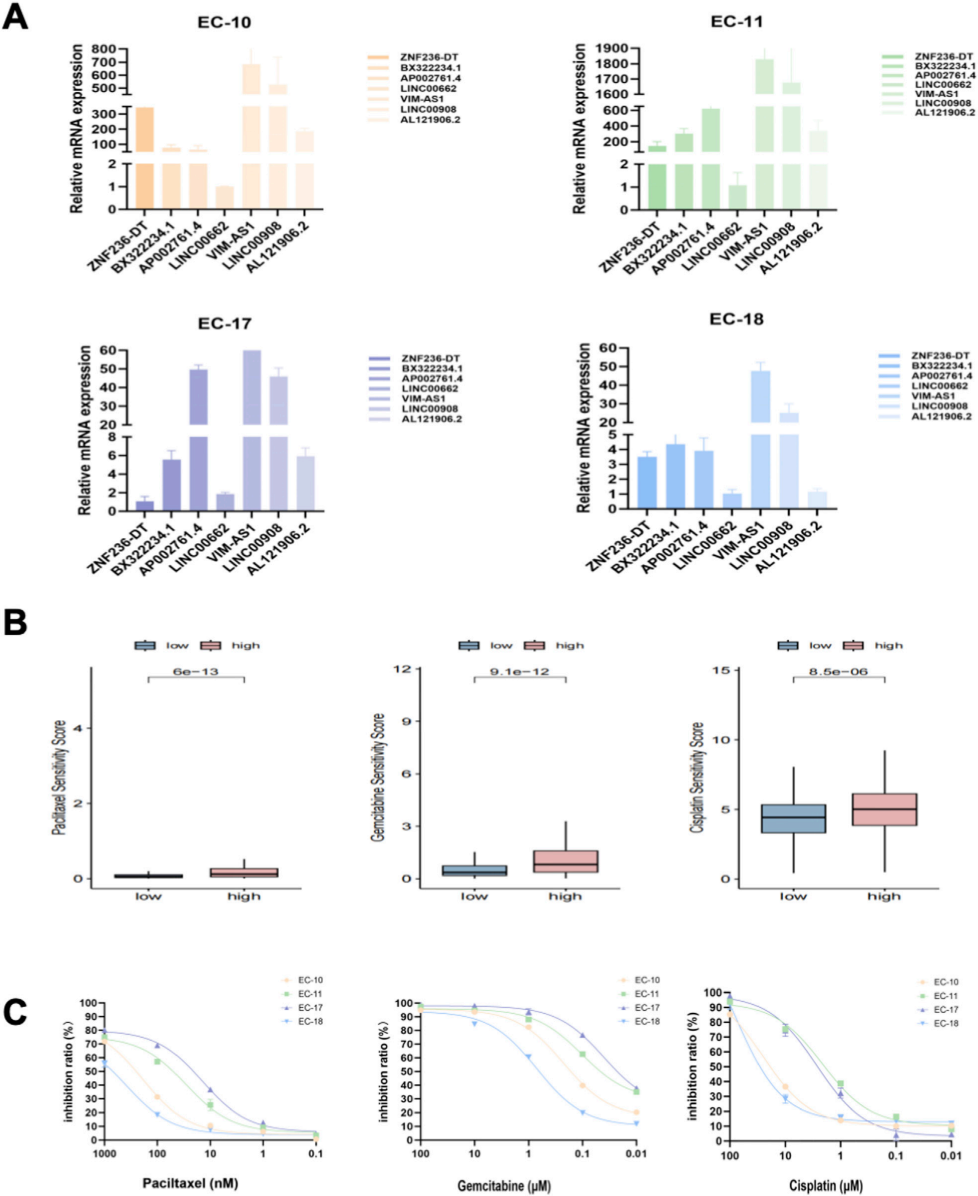
**(A)** Relative mRNA levels of targeted genes from four organoids; **(B)** Drug sensitivity analysis, including paclitaxel, gemcitabine, and cisplatin, based on the GDSC database; **(C)** The inhibition ratio plot of organoids treated with various concentrations of paclitaxel, gemcitabine, and cisplatin. GDSC, Drug Sensitivity in Cancer.

We cultured the PDOs of EC patients for drug sensitivity analysis. The morphology of EC organoids confirmed that EC organoids were successfully cultured and passaged from P0 to P2 (Figure 10A). IHC results further confirmed that the EC organoids maintained the same homological origin as their corresponding tissue (Figure 10B). Then, we calculated the relative expression of targeted srlncRNAs in tumor tissues from four EC patients. The expression levels varied significantly among different patients (Figure 11A). Subsequently, paclitaxel, gemcitabine, and cisplatin showed increased sensitivity in patients with high-risk scores (all *p* < 0.05), as indicated by the GDSC database (Figure 11B). Continually, we assessed the drug sensitivity of paclitaxel, gemcitabine, and cisplatin in EC organoids. The dose-response curves are shown in Figure 11C. The EC-18 PDO was more resistant to three drugs, which aligns with clinical observations. Our findings on drug sensitivity may help optimize individualized treatment strategies.

## Discussion

Cellular senescence is an almost universal property of biological organisms. Over the past decade, research has indicated a strong association between cellular senescence and cancers (Colucci et al., 2025). While cellular senescence is primarily viewed as an antitumor mechanism, it can also promote tumor development through interactions with the immune system (Herranz and Gil, 2018; Kowald et al., 2020; Wang et al., 2021). Recent studies have increasingly emphasized the important roles of lncRNAs in cellular senescence (Schmitt et al., 2022), the immune system (Barczak et al., 2023; Han, 2023), and the prediction of prognosis (Feng et al., 2022; Huo et al., 2022) across various cancers. However, the role of srlncRNAs in EC remains unclear. In this study, we developed a novel srlncRNA signature using machine learning analysis, which can predict survival outcomes and treatment responses for patients with EC.

Using TCGA data mining, we developed a predictive model based on seven srlncRNAs, which include AL121906.2, AP002761.4, BX322234.1, LINC00662, LINC00908, VIM-AS1, and ZNF236-DT. Among these srlncRNAs, AL121906.2 influences the prognosis of EC through glycolysis metabolism (Jiang et al., 2021). Additionally, BX322234.1 is also a prognostic biomarker for EC patients by the way of autophagy (Huo et al., 2022). Furthermore, LINC00662 has been shown to promote tumor progression in various cancers, including gallbladder cancer (Pérez-Moreno et al., 2024), osteosarcoma (Zhang et al., 2023), and melanoma (Luo et al., 2024). Similarly, LINC00908 promotes the progression of prostate cancer (Guan et al., 2025) and gastric cancer (Zhang et al., 2024). VIM-AS1 is implicated in the development of liver cancer (Han et al., 2024) and bladder cancer (Xiong et al., 2021). Lastly, AP002761.4 and ZNF236-DT appear to affect the immune microenvironment and tumor growth in acute myeloid leukemia (AML) (Zhao et al., 2024) and pancreatic adenocarcinoma (Huang et al., 2023), respectively.

In this study, ROC curves demonstrated that the predictive signature and tumor grade had better abilities in predicting survival outcomes compared to age. The tumor grade has been fully confirmed as related to the prognosis of EC patients (Tanaka et al., 2017). While our novel predictive model was the first to be established as an independent prognostic factor for EC patients. The advantage of the risk score was that it calculated each patient’s status and scored them separately, which made patient-oriented therapy possible. Moreover, it was closely related to TME and could predict the response to immunotherapy. Research indicates that patients with POLE mutations and those with mismatch-repair-deficient (dMMR) tumors tend to respond well to immunotherapy (Jamieson and McAlpine, 2023). The response rates for PD-1 inhibitors can range from 27% to 57% in advanced cases (Green et al., 2020). However, clinical observations have shown that strong responses to immunotherapy can occasionally occur in other molecular types, highlighting the necessity for more precise biomarkers to identify patients who are likely to benefit from immunotherapy. Given that the srlncRNA signature was closely related to immune infiltration in EC, we speculated that these lncRNAs could be promising targets for immune checkpoint blockade therapy.

To further validate the predictive power of our risk model in forecasting the sensitivity to commonly used chemotherapeutic drugs such as paclitaxel, gemcitabine, and cisplatin, we cultured PDOs for validation. Organoids are innovative three-dimensional, self-organizing cell cultures representing various tissues and can be used to study diverse biological systems. They provide a patient-specific model to investigate known diseases and predict treatment responses (Verstegen et al., 2025). The EC-18 PDO showed resistance to paclitaxel, gemcitabine, and cisplatin, even at high drug concentrations. This finding aligns with clinical observations, as EC patient 18 experienced disease progression quickly after chemotherapy. Therefore, we recommend that clinicians pay close attention to strategies that target the srlincRNAs identified in our model during oncology treatments. Targeting these srlncRNAs in combination with immune checkpoint inhibitors may represent a promising new approach in cancer therapy.

This study provides new insights into predicting prognosis and therapy response in EC based on a signature of srlncRNAs. Our nomogram has the potential to aid clinicians in making informed medical decisions and developing individualized treatment plans. However, our study has some limitations. First, certain factors that could influence prognosis, such as obesity, pathological types, and molecular types, were not included in the nomogram due to a lack of available data in public databases. Second, due to the lack of relevant data in the GEO and other databases, which posed challenges for external validation. We then used four organoid samples for validation. However, more samples are needed for further investigation. Lastly, functional and basic experiments are necessary to better understand the underlying mechanisms of srlncRNAs in EC.

## Conclusion

In summary, we identified a novel srlncRNA signature with prognostic predictive value. The srlncRNAs, including AL121906.2, AP002761.4, BX322234.1, LINC00662, LINC00908, VIM-AS1, and ZNF236-DT, are promising prognostic markers and potential therapeutic targets for EC patients.

## Data Availability

The data included in the current study are available in the TCGA-UCEC (https://cancergenome.nih.gov/) and the CellAge database (https://genomics.senescence.info/cells/cellAge.zip). The accession numbers are provided in Supplementary Table S7. The experiment data will be shared at a reasonable request by the corresponding author.

## Abbreviations

srlncRNA, Senescence-related lncRNAs; EC, Endometrial cancer; PDOs, Patient-derived tumor organoids; GLOBOCAN, Global Cancer Observatory; TCGA, The Cancer Genome Atlas; GBM, Boosting Machine; RSF, Survival Forest; OS, Overall survival; ROC, Receiver operating characteristic curve; GO, Gene Ontology; KEGG, Kyoto Encyclopedia of Genes and Genomes; TME, Tumor microenvironment; ssGSEA, Single-sample gene set enrichment analysis; IHC, Immunohistochemistry; GDSC, Drug Sensitivity in Cancer; IC50, Half maximal inhibitory concentration; dMMR, Mismatch-repair deficient (dMMR) tumors; AML, Acute myeloid leukemia.
