# Supplementary material for: Development of a senescence-related lncRNA signature in endometrial cancer based on multiple machine learning models
Source: Front Genet. 2025 Nov 27;16:1687922. doi: 10.3389/fgene.2025.1687922 (PMC12694935; doi:10.3389/fgene.2025.1687922)
Supplement: Supplementary file 1 [file Table1.docx]

**Table S1.** Clinical pathological parameters of EC patients in TCGA database.

| Clinical characteristics | | N | Percent |
| --- | --- | --- | --- |
| Status | Alive | 426 | 84.69% |
|  | Dead | 77 | 15.31% |
| Age | Mean (SD) | 63.9 (11.2) | - |
|  | Median [MIN, MAX] | 64 [31,90] | - |
| pTNM_stage | I | 3 | 0.60% |
|  | IA | 153 | 30.42% |
|  | IB | 134 | 26.64% |
|  | IC | 25 | 4.97% |
|  | II | 33 | 6.56% |
|  | IIA | 6 | 1.19% |
|  | IIB | 13 | 2.58% |
|  | III | 2 | 0.40% |
|  | IIIA | 34 | 6.76% |
|  | IIIB | 5 | 0.99% |
|  | IIIC | 25 | 4.97% |
|  | IIIC1 | 22 | 4.37% |
|  | IIIC2 | 21 | 4.17% |
|  | IV | 4 | 0.80% |
|  | IVA | 3 | 0.60% |
|  | IVB | 20 | 3.98% |
| Grade | G1 | 92 | 18.29% |
|  | G2 | 105 | 20.87% |
|  | G3 | 297 | 59.05% |
|  | High Grade | 9 | 1.79% |
| New tumor event | Metastasis | 37 | 7.36% |
|  | Primary | 11 | 2.19% |
|  | Recurrence | 37 | 7.36% |

**Table S2** The clinical information of EC organoids.

| Organoids | Stage | Pathological types | Size（cm） | Ki67 (%) | PTEN | MLH1 | MSH2 | MSH6 | PMS2 | P53 |
| --- | --- | --- | --- | --- | --- | --- | --- | --- | --- | --- |
| EC-10 | Ia | Endometrioid adenocarcinoma | 0.8 | 70 | - | - | + | + | - | + |
| EC-11 | Ia | Endometrioid adenocarcinoma | 2.2 | 90 | - | - | + | + | - | + |
| EC-17 | II | Endometrioid adenocarcinoma | 4.9 | / | / | + | + | - | + | + |
| EC-18 | IVb | Endometrioid adenocarcinoma | 15 | 30 | - | + | + | + | + | - |

**Table S3** Primer sequence.

| Name |  | Sequence Information（5’→ 3’） |
| --- | --- | --- |
| LINC00908 | Forward | TGACAAGAATTAAGCCAGCACAG |
|  | Reverse | TAAGGCACAGAGATGAAGGAAGT |
| VIM-AS1 | Forward | CTGGTGAGTGTTCGCTTATGAG |
|  | Reverse | ACGGTAACCTAGTGATCTGGATT |
| ZNF236-DT | Forward | CTTACACGGATCAGCAATTCAGA |
|  | Reverse | CTGTCCTCTCCAATGAAGATGTG |
| AL121906.2 | Forward | TGGCTACAAGGCAACTCAGA |
|  | Reverse | AGGCTGGTGGAAGAAACTGT |
| AP002761.4 | Forward | GCCTGACCTTAAACCCTTTCT |
|  | Reverse | GCACCCTTAGGACCTGGTTACTT |
| BX322234.1 | Forward | CGAGTTGGTTGAGCCTTGTA |
|  | Reverse | CCATCTCCCTTCTGCGAATC |
| LINC00662 | Forward | GCGGACGCAGCAGGACAGAAT |
|  | Reverse | GTTGCAGTGAGCCGAGACAGG |
| GADPH | Forward | GGTGTGAACCATGAGAAGTATGA |
|  | Reverse | GAGTCCTTCCACGATACCAAAG |

**Table S4.** Seven lncRNAs of the model and the coefficients from the multivariate Cox regression

| LncRNA | Coefficient | HR | 95% CI of HR |
| --- | --- | --- | --- |
| ZNF236-DT | -0.582 | 0.559 | 0.269-1.161 |
| BX322234.1 | 0.596 | 1.815 | 1.002-3.288 |
| AP002761.4 | 0.339 | 1.404 | 0.955-2.063 |
| AL121906.2 | 0.573 | 1.774 | 0.899-3.499 |
| VIM-AS1 | -0.425 | 0.654 | 0.388-1.101 |
| LINC00908 | -0.507 | 0.603 | 0.301-1.207 |
| LINC00662 | 0.642 | 1.900 | 0.903-3.998 |

**Table S5**. The comparison of all tested machine learning models in test set.

| Models | AUC | | |
| --- | --- | --- | --- |
|  | 1-year | 3-year | 5-year |
| GBM | 0.719, 95% IC (0.574-0.864) | 0.697, 95% IC (0.600-0.794) | 0.659, 95% IC (0.562-0.755) |
| RSF | 0.703, 95%IC (0.527-0.878) | 0.693, 95%IC (0.594-0.793) | 0.669, 95%IC (0.573-0.764) |
| CoxBoost | 0.719, 95%IC (0.503-0.935) | 0.741, 95% IC (0.647-0.835) | 0.722, 95%IC (0.627-0.816) |
| SurvivalSVM | 0.744, 95%IC (0.593-0.895) | 0.714, 95%IC (0.621-0.808) | 0.691, 95%IC (0.596-0.786) |
| Lasso regression | 0.714, 95%IC (0.515-0.916) | 0.751, 95%IC (0.658-0.860) | 0.730, 95%IC (0.606-0.845) |

AUC, The area under the curve; GBM, Gradient Boosting Machine; RFS, Random Survival Forest.

**Table S6**. The proportion of high and low risk scores across TCGA molecular subtypes.

| TCGA molecular subtypes | High risk | Low risk | χ2 | *P* |
| --- | --- | --- | --- | --- |
| CNH | 129 | 16 | 153.19 | < 0.001 |
| CNL | 27 | 107 |  |  |
| MSI | 38 | 78 |  |  |
| POLE | 29 | 45 |  |  |
| NA | 15 | 11 |  |  |

CNH, copy-number high; CNL, copy-number low; POLE, POLE ultramutated; MSI, microsatellite instability.

**Table S7** Accession numbers in TCGA

| TCGA-AX-A2HD-11A-11R-A32Y-07 |
| --- |
| TCGA-BK-A4ZD-11A-12R-A27V-07 |
| TCGA-FL-A1YH-11A-11R-A16F-07 |
| TCGA-FL-A3WE-11A-11R-A22K-07 |
| TCGA-FL-A1YG-11A-12R-A16F-07 |
| TCGA-FL-A1YI-11A-11R-A16F-07 |
| TCGA-FL-A1YU-11A-11R-A32Y-07 |
| TCGA-AJ-A2QL-11A-11R-A18M-07 |
| TCGA-AX-A2HA-11A-11R-A18M-07 |
| TCGA-AJ-A3NH-11A-11R-A22K-07 |
| TCGA-AX-A2H8-11A-11R-A17B-07 |
| TCGA-FL-A1YM-11A-12R-A17B-07 |
| TCGA-AX-A2HC-11A-11R-A32Y-07 |
| TCGA-FL-A1YN-11A-11R-A32Y-07 |
| TCGA-AX-A1CF-11A-11R-A137-07 |
| TCGA-AX-A1CK-11A-11R-A137-07 |
| TCGA-BG-A3PP-11A-11R-A22K-07 |
| TCGA-BG-A3EW-11A-22R-A22K-07 |
| TCGA-FL-A1YF-11A-12R-A16F-07 |
| TCGA-DI-A2QY-11A-11R-A19W-07 |
| TCGA-AJ-A3NE-11A-11R-A22K-07 |
| TCGA-AX-A0J0-11A-11R-A27V-07 |
| TCGA-AX-A1CI-11A-11R-A137-07 |
| TCGA-BG-A2AD-11A-11R-A16F-07 |
| TCGA-FL-A1YT-11A-12R-A32Y-07 |
| TCGA-FL-A1YL-11A-11R-A16F-07 |
| TCGA-AX-A05Y-11A-11R-A27V-07 |
| TCGA-FL-A1YV-11A-12R-A32Y-07 |
| TCGA-AJ-A3NC-11A-11R-A22K-07 |
| TCGA-AX-A0IZ-11A-11R-A27V-07 |
| TCGA-BK-A0CB-11A-33R-A104-07 |
| TCGA-DI-A2QU-11A-11R-A18M-07 |
| TCGA-E6-A1M0-11A-11R-A144-07 |
| TCGA-BK-A13C-11A-11R-A118-07 |
| TCGA-FL-A1YQ-11A-11R-A32Y-07 |
| TCGA-D1-A101-01A-12R-A10J-07 |
| TCGA-EY-A72D-01A-12R-A34R-07 |
| TCGA-D1-A2G6-01A-11R-A17B-07 |
| TCGA-PG-A916-01A-11R-A37O-07 |
| TCGA-AJ-A3OK-01A-12R-A22K-07 |
| TCGA-A5-A7WK-01A-11R-A34R-07 |
| TCGA-D1-A179-01A-11R-A12I-07 |
| TCGA-QS-A8F1-01A-21R-A37O-07 |
| TCGA-AX-A05T-01A-11R-A00V-07 |
| TCGA-AJ-A3NH-01A-11R-A22K-07 |
| TCGA-AX-A3FW-01A-11R-A22K-07 |
| TCGA-AP-A0LV-01A-11R-A040-07 |
| TCGA-AJ-A2QM-01A-11R-A18M-07 |
| TCGA-B5-A5OD-01A-11R-A31O-07 |
| TCGA-A5-A0GA-01A-11R-A040-07 |
| TCGA-KJ-A3U4-01A-11R-A22K-07 |
| TCGA-B5-A0K2-01A-12R-A104-07 |
| TCGA-D1-A17T-01A-11R-A12I-07 |
| TCGA-D1-A16J-01A-11R-A12I-07 |
| TCGA-FI-A2D5-01A-11R-A17B-07 |
| TCGA-AP-A0LD-01A-11R-A040-07 |
| TCGA-D1-A162-01A-11R-A118-07 |
| TCGA-AP-A0LS-01A-11R-A14D-07 |
| TCGA-B5-A1MV-01A-31R-A14D-07 |
| TCGA-D1-A17L-01A-11R-A12I-07 |
| TCGA-EO-A22U-01A-11R-A180-07 |
| TCGA-AP-A1DK-01A-11R-A137-07 |
| TCGA-A5-A0R9-01A-11R-A104-07 |
| TCGA-B5-A11J-01A-11R-A118-07 |
| TCGA-BS-A0UT-01A-11R-A104-07 |
| TCGA-AP-A0LL-01A-12R-A104-07 |
| TCGA-B5-A1MS-01B-11R-A22K-07 |
| TCGA-D1-A16X-01A-11R-A12I-07 |
| TCGA-AX-A064-01A-11R-A00V-07 |
| TCGA-AJ-A3NF-01A-11R-A22K-07 |
| TCGA-QS-A744-01A-11R-A34R-07 |
| TCGA-QF-A5YS-01A-11R-A31O-07 |
| TCGA-D1-A167-01A-11R-A12I-07 |
| TCGA-DF-A2KS-01A-11R-A18M-07 |
| TCGA-BG-A3EW-01A-11R-A22K-07 |
| TCGA-AX-A2HC-01A-11R-A17B-07 |
| TCGA-A5-A0VP-01A-21R-A104-07 |
| TCGA-A5-A0GP-01A-11R-A040-07 |
| TCGA-AX-A1C9-01A-11R-A137-07 |
| TCGA-BG-A0W1-01A-12R-A109-07 |
| TCGA-A5-A0G1-01A-11R-A118-07 |
| TCGA-EY-A1G7-01A-11R-A13S-07 |
| TCGA-D1-A0ZP-01A-21R-A10J-07 |
| TCGA-BS-A0VI-01A-11R-A14D-07 |
| TCGA-AX-A3FT-01A-11R-A22K-07 |
| TCGA-D1-A3DG-01A-11R-A19W-07 |
| TCGA-BG-A0RY-01A-11R-A104-07 |
| TCGA-D1-A1O0-01A-11R-A17B-07 |
| TCGA-AJ-A3OJ-01A-11R-A22K-07 |
| TCGA-D1-A177-01A-21R-A12I-07 |
| TCGA-EY-A1GX-01A-12R-A13S-07 |
| TCGA-A5-A2K5-01A-11R-A180-07 |
| TCGA-D1-A15W-01A-11R-A118-07 |
| TCGA-DI-A1BU-01A-11R-A137-07 |
| TCGA-BS-A0TG-01A-32R-A16W-07 |
| TCGA-BG-A0VX-01A-11R-A118-07 |
| TCGA-D1-A160-01A-11R-A118-07 |
| TCGA-AX-A3G7-01A-12R-A213-07 |
| TCGA-EY-A1GE-01A-11R-A13S-07 |
| TCGA-B5-A11F-01A-11R-A10J-07 |
| TCGA-BK-A6W3-01A-12R-A34R-07 |
| TCGA-D1-A15V-01A-11R-A118-07 |
| TCGA-AX-A063-01A-11R-A00V-07 |
| TCGA-AX-A2H2-01A-11R-A180-07 |
| TCGA-B5-A11L-01B-21R-A13S-07 |
| TCGA-EY-A1G8-01A-11R-A13S-07 |
| TCGA-D1-A102-01A-11R-A10J-07 |
| TCGA-D1-A3DA-01A-12R-A213-07 |
| TCGA-BG-A220-01A-11R-A157-07 |
| TCGA-AP-A051-01A-21R-A00V-07 |
| TCGA-D1-A17D-01A-12R-A12I-07 |
| TCGA-SJ-A6ZJ-01A-12R-A34R-07 |
| TCGA-BG-A0MG-01A-21R-A104-07 |
| TCGA-BK-A0CB-01A-32R-A104-07 |
| TCGA-D1-A103-01A-11R-A10J-07 |
| TCGA-D1-A17B-01A-22R-A12I-07 |
| TCGA-AX-A1CE-01A-11R-A137-07 |
| TCGA-A5-A0R7-01A-31R-A16W-07 |
| TCGA-DF-A2L0-01A-11R-A180-07 |
| TCGA-D1-A16G-01A-31R-A12I-07 |
| TCGA-EY-A1GK-01A-11R-A13S-07 |
| TCGA-D1-A165-01A-11R-A12I-07 |
| TCGA-B5-A0JS-01A-11R-A104-07 |
| TCGA-BS-A0U5-01A-11R-A109-07 |
| TCGA-D1-A1NY-01A-11R-A16F-07 |
| TCGA-BK-A139-01A-11R-A277-07 |
| TCGA-B5-A1MW-01A-11R-A32Y-07 |
| TCGA-PG-A917-01A-31R-A37O-07 |
| TCGA-5S-A9Q8-01A-11R-A40A-07 |
| TCGA-B5-A1N2-01A-21R-A144-07 |
| TCGA-A5-A1OG-01A-11R-A14D-07 |
| TCGA-BG-A0M0-01A-11R-A104-07 |
| TCGA-AX-A3FZ-01A-11R-A22K-07 |
| TCGA-BS-A0UV-01A-11R-A16W-07 |
| TCGA-EY-A1GQ-01A-21R-A13S-07 |
| TCGA-B5-A3FC-01A-11R-A22K-07 |
| TCGA-D1-A16R-01A-11R-A12I-07 |
| TCGA-AJ-A5DV-01A-11R-A27V-07 |
| TCGA-A5-A2K7-01A-11R-A180-07 |
| TCGA-AJ-A3IA-01A-11R-A213-07 |
| TCGA-EY-A4KR-01A-11R-A27V-07 |
| TCGA-AP-A0LP-01A-12R-A104-07 |
| TCGA-B5-A0K3-01A-11R-A040-07 |
| TCGA-BG-A0YU-01A-21R-A10J-07 |
| TCGA-DF-A2KZ-01A-11R-A213-07 |
| TCGA-EY-A210-01A-11R-A157-07 |
| TCGA-FI-A2F8-01A-12R-A17B-07 |
| TCGA-EY-A548-01A-11R-A27V-07 |
| TCGA-SL-A6J9-01A-11R-A31O-07 |
| TCGA-AJ-A23M-01A-11R-A157-07 |
| TCGA-B5-A0JR-01A-13R-A466-07 |
| TCGA-B5-A3FD-01A-11R-A19W-07 |
| TCGA-E6-A2P9-01A-11R-A19W-07 |
| TCGA-QS-A5YQ-01A-11R-A31O-07 |
| TCGA-AX-A1CC-01A-11R-A137-07 |
| TCGA-EY-A1GT-01A-11R-A13S-07 |
| TCGA-A5-A0GH-01A-21R-A16W-07 |
| TCGA-EO-A22Y-01A-11R-A180-07 |
| TCGA-AJ-A3QS-01A-11R-A22K-07 |
| TCGA-A5-A0GW-01A-11R-A16W-07 |
| TCGA-AP-A0LH-01A-11R-A16W-07 |
| TCGA-EY-A3QX-01A-11R-A22K-07 |
| TCGA-AJ-A2QO-01A-11R-A32Y-07 |
| TCGA-AP-A052-01A-11R-A00V-07 |
| TCGA-D1-A1NW-01A-11R-A14M-07 |
| TCGA-AP-A1DO-01A-11R-A137-07 |
| TCGA-D1-A3JP-01A-31R-A22K-07 |
| TCGA-AX-A2IO-01A-11R-A180-07 |
| TCGA-EO-A1Y8-01A-11R-A157-07 |
| TCGA-AX-A3G1-01A-11R-A22K-07 |
| TCGA-D1-A16Y-01A-31R-A12I-07 |
| TCGA-B5-A0JZ-01A-11R-A040-07 |
| TCGA-PG-A7D5-01A-11R-A34R-07 |
| TCGA-AX-A1C5-01A-11R-A137-07 |
| TCGA-AP-A5FX-01A-11R-A27V-07 |
| TCGA-EY-A1GD-01A-11R-A13S-07 |
| TCGA-AX-A0IS-01A-12R-A10J-07 |
| TCGA-D1-A0ZV-01A-11R-A10J-07 |
| TCGA-D1-A0ZN-01A-11R-A118-07 |
| TCGA-B5-A11P-01B-11R-A14D-07 |
| TCGA-AJ-A8CV-01A-11R-A37O-07 |
| TCGA-BG-A0MA-01A-11R-A17B-07 |
| TCGA-B5-A0K0-01A-11R-A16W-07 |
| TCGA-BG-A3PP-01A-11R-A22K-07 |
| TCGA-A5-A0G5-01A-11R-A040-07 |
| TCGA-AX-A1C7-01A-11R-A137-07 |
| TCGA-A5-A0GQ-01A-11R-A118-07 |
| TCGA-D1-A174-01A-11R-A12I-07 |
| TCGA-BS-A0UA-01A-11R-A118-07 |
| TCGA-AX-A2HA-01A-12R-A18M-07 |
| TCGA-EY-A547-01A-11R-A27V-07 |
| TCGA-E6-A2P8-01A-11R-A19W-07 |
| TCGA-AX-A05Y-01A-11R-A00V-07 |
| TCGA-B5-A0K1-01A-11R-A16W-07 |
| TCGA-BK-A26L-01A-11R-A277-07 |
| TCGA-BS-A0TC-01A-11R-A104-07 |
| TCGA-B5-A11U-01A-11R-A118-07 |
| TCGA-EO-A3AZ-01A-12R-A19W-07 |
| TCGA-A5-A0GX-01A-11R-A040-07 |
| TCGA-E6-A1M0-01A-11R-A144-07 |
| TCGA-BS-A0U7-01A-21R-A104-07 |
| TCGA-D1-A16V-01A-11R-A12I-07 |
| TCGA-AX-A1C8-01A-11R-A137-07 |
| TCGA-AJ-A3NG-01A-11R-A22K-07 |
| TCGA-AP-A0LI-01A-11R-A040-07 |
| TCGA-A5-A0GI-01A-11R-A040-07 |
| TCGA-B5-A3FH-01A-11R-A19W-07 |
| TCGA-AP-A056-01A-11R-A00V-07 |
| TCGA-AP-A0LT-01A-11R-A040-07 |
| TCGA-AP-A0LN-01A-11R-A040-07 |
| TCGA-E6-A1LX-01A-11R-A14D-07 |
| TCGA-BS-A0WQ-01A-21R-A109-07 |
| TCGA-AP-A0LM-01A-11R-A118-07 |
| TCGA-D1-A15Z-01A-11R-A118-07 |
| TCGA-AJ-A2QK-01A-11R-A18M-07 |
| TCGA-AP-A3K1-01A-11R-A213-07 |
| TCGA-B5-A3F9-01A-21R-A22K-07 |
| TCGA-KP-A3W0-01A-21R-A22K-07 |
| TCGA-AJ-A8CW-01A-11R-A37O-07 |
| TCGA-BK-A4ZD-01A-11R-A27V-07 |
| TCGA-KP-A3W3-01A-11R-A22K-07 |
| TCGA-BG-A0M2-01A-11R-A104-07 |
| TCGA-D1-A1O5-01A-11R-A14D-07 |
| TCGA-AX-A3FS-01A-11R-A22K-07 |
| TCGA-BG-A0MH-01A-11R-A118-07 |
| TCGA-A5-A0GG-01A-11R-A118-07 |
| TCGA-PG-A914-01A-11R-A37O-07 |
| TCGA-B5-A11M-01A-11R-A118-07 |
| TCGA-EO-A3L0-01A-11R-A22K-07 |
| TCGA-AJ-A3OL-01A-11R-A22K-07 |
| TCGA-DI-A2QU-01A-11R-A18M-07 |
| TCGA-AX-A05S-01A-11R-A00V-07 |
| TCGA-AX-A05W-01A-21R-A466-07 |
| TCGA-AX-A05U-01A-11R-A00V-07 |
| TCGA-BS-A0TA-01A-11R-A104-07 |
| TCGA-BG-A0MS-01A-11R-A104-07 |
| TCGA-A5-A0RA-01A-21R-A104-07 |
| TCGA-EY-A1GS-01A-11R-A13S-07 |
| TCGA-AP-A1DR-01A-11R-A137-07 |
| TCGA-AX-A2H5-01A-11R-A17B-07 |
| TCGA-D1-A0ZZ-01A-11R-A10J-07 |
| TCGA-FI-A2EX-01A-11R-A17B-07 |
| TCGA-D1-A16F-01A-11R-A12I-07 |
| TCGA-AJ-A3BD-01A-11R-A19W-07 |
| TCGA-BG-A0VT-01A-11R-A10J-07 |
| TCGA-QS-A5YR-01A-31R-A31O-07 |
| TCGA-BG-A0MI-01A-11R-A040-07 |
| TCGA-EY-A1GW-01A-22R-A13S-07 |
| TCGA-EO-A22T-01A-21R-A18M-07 |
| TCGA-B5-A1MX-01A-11R-A144-07 |
| TCGA-AX-A2H4-01A-21R-A18M-07 |
| TCGA-A5-A0GE-01A-11R-A16W-07 |
| TCGA-AJ-A3EL-01A-11R-A213-07 |
| TCGA-EO-A22R-01A-11R-A18M-07 |
| TCGA-AX-A2HD-01A-21R-A17B-07 |
| TCGA-A5-A1OH-01A-21R-A22K-07 |
| TCGA-D1-A0ZQ-01A-11R-A118-07 |
| TCGA-AP-A1E1-01A-11R-A137-07 |
| TCGA-BG-A0M7-01A-11R-A040-07 |
| TCGA-FI-A2CX-01A-11R-A17B-07 |
| TCGA-AX-A2IN-01A-12R-A180-07 |
| TCGA-EY-A1H0-01A-11R-A13S-07 |
| TCGA-EY-A1GU-01A-11R-A13S-07 |
| TCGA-D1-A2G7-01A-21R-A180-07 |
| TCGA-B5-A0K8-01A-11R-A14M-07 |
| TCGA-DF-A2KU-01A-11R-A180-07 |
| TCGA-AP-A1E4-01A-12R-A137-07 |
| TCGA-B5-A11W-01A-12R-A118-07 |
| TCGA-BG-A0LX-01A-11R-A16W-07 |
| TCGA-EY-A549-01A-11R-A27V-07 |
| TCGA-D1-A17M-01A-21R-A12I-07 |
| TCGA-D1-A1NZ-01A-21R-A14D-07 |
| TCGA-D1-A17H-01A-11R-A12I-07 |
| TCGA-AP-A1E0-01A-11R-A137-07 |
| TCGA-B5-A0JT-01A-21R-A118-07 |
| TCGA-FI-A2CY-01A-11R-A17B-07 |
| TCGA-PG-A915-01A-11R-A37O-07 |
| TCGA-EC-A24G-01A-11R-A16F-07 |
| TCGA-PG-A5BC-01A-12R-A27V-07 |
| TCGA-AJ-A3BI-01A-11R-A213-07 |
| TCGA-D1-A16B-01A-11R-A12I-07 |
| TCGA-BG-A2AE-01A-11R-A16F-07 |
| TCGA-A5-A0G2-01A-11R-A16W-07 |
| TCGA-D1-A176-01A-11R-A12I-07 |
| TCGA-EY-A2ON-01A-21R-A18M-07 |
| TCGA-BG-A221-01A-21R-A157-07 |
| TCGA-AP-A0LJ-01A-11R-A040-07 |
| TCGA-AX-A06F-01A-11R-A00V-07 |
| TCGA-AX-A0J0-01A-11R-A109-07 |
| TCGA-AX-A060-01A-11R-A00V-07 |
| TCGA-D1-A17Q-01A-11R-A12I-07 |
| TCGA-BG-A222-01A-11R-A157-07 |
| TCGA-B5-A11N-01A-11R-A118-07 |
| TCGA-BK-A6W4-01A-12R-A34R-07 |
| TCGA-B5-A11E-01A-11R-A10J-07 |
| TCGA-BG-A0W2-01A-11R-A109-07 |
| TCGA-AP-A053-01A-21R-A00V-07 |
| TCGA-D1-A3JQ-01A-11R-A22K-07 |
| TCGA-D1-A16D-01A-11R-A12I-07 |
| TCGA-B5-A11R-01A-11R-A118-07 |
| TCGA-B5-A11Q-01A-11R-A118-07 |
| TCGA-QF-A5YT-01A-11R-A31O-07 |
| TCGA-AP-A054-01A-11R-A16W-07 |
| TCGA-B5-A11S-01A-11R-A118-07 |
| TCGA-AX-A3GI-01A-11R-A213-07 |
| TCGA-B5-A0JY-01A-11R-A104-07 |
| TCGA-BK-A26L-01C-04R-A277-07 |
| TCGA-A5-A1OJ-01A-11R-A14D-07 |
| TCGA-AX-A2HJ-01A-11R-A17B-07 |
| TCGA-BK-A26L-01A-11R-A16F-07 |
| TCGA-FI-A2F9-01A-11R-A17B-07 |
| TCGA-AX-A06D-01A-11R-A118-07 |
| TCGA-BS-A0UM-01A-11R-A104-07 |
| TCGA-EY-A2OM-01A-11R-A18M-07 |
| TCGA-B5-A0JU-01B-11R-A14D-07 |
| TCGA-AX-A1CK-01A-11R-A137-07 |
| TCGA-EY-A1GM-01A-12R-A14D-07 |
| TCGA-B5-A1MU-01A-11R-A13S-07 |
| TCGA-B5-A11O-01A-11R-A118-07 |
| TCGA-BK-A139-01A-11R-A118-07 |
| TCGA-AJ-A3BH-01A-11R-A19W-07 |
| TCGA-KP-A3W1-01A-11R-A22K-07 |
| TCGA-B5-A11I-01A-11R-A10J-07 |
| TCGA-D1-A163-01A-11R-A12I-07 |
| TCGA-B5-A11Y-01A-21R-A10J-07 |
| TCGA-A5-A0GR-01A-11R-A118-07 |
| TCGA-BK-A0CC-01A-21R-A277-07 |
| TCGA-B5-A0JV-01A-11R-A104-07 |
| TCGA-B5-A3FA-01A-11R-A19W-07 |
| TCGA-D1-A1NX-01A-11R-A16F-07 |
| TCGA-H5-A2HR-01A-11R-A180-07 |
| TCGA-EY-A1GP-01A-11R-A13S-07 |
| TCGA-EY-A215-01A-11R-A14M-07 |
| TCGA-BG-A186-01A-11R-A12I-07 |
| TCGA-BK-A0CC-01B-04R-A277-07 |
| TCGA-D1-A16I-01A-11R-A12I-07 |
| TCGA-A5-A0R6-01A-11R-A104-07 |
| TCGA-B5-A1MZ-01A-11R-A144-07 |
| TCGA-A5-A0R8-01A-11R-A104-07 |
| TCGA-EY-A1GL-01A-11R-A13S-07 |
| TCGA-B5-A11V-01A-11R-A10J-07 |
| TCGA-A5-A1OK-01A-11R-A14M-07 |
| TCGA-B5-A0JN-01A-11R-A104-07 |
| TCGA-FI-A2EU-01A-11R-A17B-07 |
| TCGA-5B-A90C-01A-11R-A37O-07 |
| TCGA-EY-A212-01A-11R-A14M-07 |
| TCGA-PG-A6IB-01A-21R-A31O-07 |
| TCGA-AP-A05O-01A-12R-A118-07 |
| TCGA-AX-A2HK-01A-11R-A17B-07 |
| TCGA-AP-A1DQ-01A-11R-A137-07 |
| TCGA-BG-A0MU-01A-11R-A104-07 |
| TCGA-EO-A3KW-01A-11R-A22K-07 |
| TCGA-DF-A2KN-01A-11R-A180-07 |
| TCGA-BK-A56F-01A-32R-A27V-07 |
| TCGA-A5-A1OF-01A-11R-A14D-07 |
| TCGA-D1-A17U-01A-21R-A12I-07 |
| TCGA-B5-A11Z-01A-11R-A10J-07 |
| TCGA-SL-A6JA-01A-11R-A31O-07 |
| TCGA-B5-A3S1-01A-11R-A22K-07 |
| TCGA-AX-A2HG-01A-11R-A17B-07 |
| TCGA-AJ-A3EK-01A-11R-A19W-07 |
| TCGA-A5-A2K2-01A-11R-A18M-07 |
| TCGA-A5-A0GV-01A-31R-A16W-07 |
| TCGA-EO-A22S-01A-11R-A18M-07 |
| TCGA-FI-A2EY-01A-12R-A18M-07 |
| TCGA-A5-A7WJ-01A-12R-A34R-07 |
| TCGA-JU-AAVI-01A-11R-A40A-07 |
| TCGA-AJ-A3BK-01A-11R-A19W-07 |
| TCGA-AX-A3FV-01A-11R-A22K-07 |
| TCGA-AJ-A3TW-01A-11R-A22K-07 |
| TCGA-D1-A0ZO-01A-11R-A118-07 |
| TCGA-A5-A3LP-01A-11R-A22K-07 |
| TCGA-D1-A17C-01A-11R-A12I-07 |
| TCGA-BK-A13C-01A-11R-A118-07 |
| TCGA-EO-A3KX-01A-11R-A22K-07 |
| TCGA-B5-A0K4-01A-11R-A040-07 |
| TCGA-K6-A3WQ-01A-11R-A22K-07 |
| TCGA-DF-A2KY-01A-21R-A213-07 |
| TCGA-AP-A1DH-01A-31R-A137-07 |
| TCGA-D1-A17F-01A-11R-A12I-07 |
| TCGA-D1-A168-01A-31R-A12I-07 |
| TCGA-A5-AB3J-01A-11R-A40A-07 |
| TCGA-DF-A2KV-01A-11R-A180-07 |
| TCGA-EO-A1Y5-01A-11R-A157-07 |
| TCGA-B5-A0K9-01A-21R-A104-07 |
| TCGA-E6-A1LZ-01A-11R-A144-07 |
| TCGA-KP-A3W4-01A-11R-A22K-07 |
| TCGA-AX-A3GB-01A-11R-A22K-07 |
| TCGA-BS-A0V4-01A-11R-A14D-07 |
| TCGA-AX-A3FX-01A-11R-A22K-07 |
| TCGA-BG-A0M9-01A-21R-A104-07 |
| TCGA-D1-A16N-01A-11R-A12I-07 |
| TCGA-B5-A11G-01A-13R-A118-07 |
| TCGA-AP-A1DM-01A-21R-A137-07 |
| TCGA-DI-A1NN-01A-11R-A16F-07 |
| TCGA-AX-A3G4-01A-11R-A213-07 |
| TCGA-AX-A0J1-01A-11R-A040-07 |
| TCGA-AJ-A23N-01A-11R-A22K-07 |
| TCGA-B5-A121-01A-31R-A118-07 |
| TCGA-AJ-A3BF-01A-11R-A213-07 |
| TCGA-A5-A3LO-01A-11R-A22K-07 |
| TCGA-AP-A059-01A-21R-A118-07 |
| TCGA-FI-A2F4-01A-11R-A17B-07 |
| TCGA-AP-A1DV-01A-21R-A137-07 |
| TCGA-BS-A0T9-01A-11R-A12I-07 |
| TCGA-EO-A3AS-01A-11R-A19W-07 |
| TCGA-D1-A1O7-01A-11R-A14D-07 |
| TCGA-D1-A17K-01A-11R-A12I-07 |
| TCGA-AP-A0LF-01A-11R-A118-07 |
| TCGA-A5-A2K4-01A-11R-A18M-07 |
| TCGA-BG-A0MO-01A-11R-A040-07 |
| TCGA-FI-A2D6-01A-11R-A17B-07 |
| TCGA-AJ-A3I9-01A-11R-A22K-07 |
| TCGA-AP-A0LO-01A-11R-A040-07 |
| TCGA-AJ-A3EJ-01A-11R-A19W-07 |
| TCGA-B5-A0K7-01A-11R-A104-07 |
| TCGA-BG-A2L7-01A-11R-A18M-07 |
| TCGA-AX-A062-01A-11R-A00V-07 |
| TCGA-FI-A2D2-01A-11R-A17B-07 |
| TCGA-EY-A214-01A-12R-A157-07 |
| TCGA-B5-A5OE-01A-11R-A31O-07 |
| TCGA-AJ-A2QL-01A-11R-A18M-07 |
| TCGA-DI-A0WH-01A-12R-A12I-07 |
| TCGA-AX-A1CF-01A-11R-A137-07 |
| TCGA-BS-A0TJ-01A-11R-A104-07 |
| TCGA-D1-A15X-01A-11R-A118-07 |
| TCGA-EO-A3B1-01A-12R-A19W-07 |
| TCGA-BK-A0CA-01B-02R-A277-07 |
| TCGA-AX-A3G8-01A-11R-A22K-07 |
| TCGA-D1-A1NS-01A-11R-A14D-07 |
| TCGA-AJ-A3NC-01A-11R-A22K-07 |
| TCGA-AX-A0IW-01A-11R-A040-07 |
| TCGA-AP-A05A-01A-11R-A00V-07 |
| TCGA-AJ-A8CT-01A-11R-A37O-07 |
| TCGA-BG-A18C-01A-11R-A12I-07 |
| TCGA-BS-A0UF-01A-11R-A104-07 |
| TCGA-SJ-A6ZI-01A-12R-A34R-07 |
| TCGA-BS-A0V7-01A-21R-A118-07 |
| TCGA-AX-A0IZ-01A-11R-A118-07 |
| TCGA-EY-A1GC-01A-11R-A13S-07 |
| TCGA-A5-A0VO-01A-21R-A109-07 |
| TCGA-EY-A1GR-01A-11R-A13S-07 |
| TCGA-BK-A0CA-01A-21R-A118-07 |
| TCGA-EY-A1GI-01A-11R-A13S-07 |
| TCGA-EC-A1QX-01A-31R-A16F-07 |
| TCGA-E6-A8L9-01A-21R-A37O-07 |
| TCGA-B5-A3FB-01A-11R-A19W-07 |
| TCGA-BG-A18B-01A-11R-A12I-07 |
| TCGA-4E-A92E-01A-11R-A37O-07 |
| TCGA-EY-A1GV-01A-11R-A13S-07 |
| TCGA-B5-A0K6-01A-11R-A040-07 |
| TCGA-BS-A0TI-01A-11R-A104-07 |
| TCGA-BG-A18A-01A-21R-A12I-07 |
| TCGA-BS-A0U8-01A-11R-A104-07 |
| TCGA-BK-A13B-01A-51R-A22K-07 |
| TCGA-AX-A2HF-01A-11R-A17B-07 |
| TCGA-AX-A06H-01A-11R-A118-07 |
| TCGA-AW-A1PO-01A-12R-A157-07 |
| TCGA-EY-A2OO-01A-11R-A19W-07 |
| TCGA-AX-A1CJ-01A-11R-A137-07 |
| TCGA-AX-A1CI-01A-11R-A137-07 |
| TCGA-AX-A05Z-01A-11R-A00V-07 |
| TCGA-BS-A0V8-01A-11R-A118-07 |
| TCGA-A5-A2K3-01A-11R-A32Y-07 |
| TCGA-AX-A3G6-01A-11R-A213-07 |
| TCGA-D1-A17S-01A-11R-A12I-07 |
| TCGA-AX-A1CN-01A-11R-A137-07 |
| TCGA-D1-A17R-01A-11R-A12I-07 |
| TCGA-BS-A0U9-01B-21R-A10J-07 |
| TCGA-BG-A2AD-01A-21R-A16F-07 |
| TCGA-AX-A3G9-01A-11R-A22K-07 |
| TCGA-D1-A17N-01A-11R-A12I-07 |
| TCGA-BK-A139-01C-08R-A277-07 |
| TCGA-AP-A1DP-01A-11R-A137-07 |
| TCGA-EO-A22X-01A-11R-A180-07 |
| TCGA-AP-A05D-01A-11R-A00V-07 |
| TCGA-AP-A05P-01A-11R-A16W-07 |
| TCGA-EY-A3L3-01A-11R-A22K-07 |
| TCGA-AX-A2HH-01A-11R-A32Y-07 |
| TCGA-AJ-A3NE-01A-11R-A22K-07 |
| TCGA-BG-A0YV-01A-11R-A10J-07 |
| TCGA-D1-A16E-01A-22R-A12I-07 |
| TCGA-AJ-A23O-01A-11R-A157-07 |
| TCGA-EO-A3AY-01A-12R-A19W-07 |
| TCGA-FI-A2D0-01A-11R-A17B-07 |
| TCGA-BG-A0M4-01A-11R-A104-07 |
| TCGA-FI-A2D4-01A-12R-A17B-07 |
| TCGA-D1-A16S-01A-11R-A12I-07 |
| TCGA-A5-A0GN-01A-11R-A040-07 |
| TCGA-BG-A0VV-01A-21R-A118-07 |
| TCGA-BK-A0CC-01A-21R-A16W-07 |
| TCGA-D1-A16Q-01A-12R-A12I-07 |
| TCGA-BK-A139-02A-11R-A27V-07 |
| TCGA-BG-A187-01A-11R-A12I-07 |
| TCGA-EO-A3B0-01A-12R-A19W-07 |
| TCGA-DI-A1BY-01A-21R-A137-07 |
| TCGA-D1-A2G5-01A-11R-A17B-07 |
| TCGA-BG-A0M3-01A-11R-A104-07 |
| TCGA-EY-A5W2-01A-11R-A31O-07 |
| TCGA-D1-A1O8-01A-11R-A14D-07 |
| TCGA-DI-A2QT-01A-12R-A19W-07 |
| TCGA-FI-A3PX-01A-11R-A22K-07 |
| TCGA-D1-A1NU-01A-11R-A14D-07 |
| TCGA-AX-A2H8-01A-11R-A17B-07 |
| TCGA-DI-A2QY-01A-12R-A19W-07 |
| TCGA-BG-A0M8-01A-12R-A104-07 |
| TCGA-EO-A3AV-01A-12R-A19W-07 |
| TCGA-D1-A0ZU-01A-11R-A10J-07 |
| TCGA-AP-A0L9-01A-11R-A040-07 |
| TCGA-AX-A1CA-01A-12R-A137-07 |
| TCGA-EY-A1GF-01A-11R-A13S-07 |
| TCGA-EO-A1Y7-01A-11R-A157-07 |
| TCGA-EY-A54A-01A-11R-A27V-07 |
| TCGA-EY-A2OP-01A-11R-A19W-07 |
| TCGA-BG-A0M6-01A-31R-A104-07 |
| TCGA-BK-A0C9-01A-11R-A00V-07 |
| TCGA-DI-A1NO-01A-31R-A157-07 |
| TCGA-B5-A11X-01A-11R-A10J-07 |
| TCGA-B5-A1MR-01A-31R-A14D-07 |
| TCGA-AX-A06J-01A-11R-A00V-07 |
| TCGA-KP-A3VZ-01A-11R-A22K-07 |
| TCGA-AJ-A3EM-01A-11R-A213-07 |
| TCGA-AP-A05N-01A-11R-A00V-07 |
| TCGA-AP-A1E3-01A-11R-A137-07 |
| TCGA-EO-A2CG-01A-12R-A180-07 |
| TCGA-AX-A3G3-01A-11R-A213-07 |
| TCGA-A5-A0GU-01A-11R-A16W-07 |
| TCGA-BS-A0V6-01A-11R-A118-07 |
| TCGA-AP-A0LG-01A-11R-A16W-07 |
| TCGA-B5-A0KB-01B-11R-A14D-07 |
| TCGA-AJ-A3BG-01A-11R-A19W-07 |
| TCGA-EC-A1NJ-01A-31R-A14D-07 |
| TCGA-AX-A0IU-01A-11R-A16W-07 |
| TCGA-AX-A06L-01A-11R-A118-07 |
| TCGA-D1-A175-01A-11R-A12I-07 |
| TCGA-D1-A169-01A-11R-A12I-07 |
| TCGA-A5-A0G3-01A-11R-A040-07 |
| TCGA-AX-A1CR-01A-12R-A137-07 |
| TCGA-DF-A2KR-01A-11R-A180-07 |
| TCGA-A5-A0VQ-01A-11R-A104-07 |
| TCGA-B5-A1MY-01A-11R-A144-07 |
| TCGA-D1-A161-01A-11R-A118-07 |
| TCGA-BG-A0MQ-01A-11R-A104-07 |
| TCGA-BG-A0MC-01A-21R-A040-07 |
| TCGA-AX-A1CP-01A-11R-A137-07 |
| TCGA-FI-A3PV-01A-11R-A22K-07 |
| TCGA-EY-A1GO-01A-11R-A14D-07 |
| TCGA-EY-A1GH-01A-11R-A13S-07 |
| TCGA-A5-A0GD-01A-11R-A16W-07 |
| TCGA-EO-A3KU-01A-11R-A22K-07 |
| TCGA-D1-A16O-01A-11R-A12I-07 |
| TCGA-EO-A2CH-01A-11R-A180-07 |
| TCGA-D1-A0ZS-01A-11R-A118-07 |
| TCGA-B5-A5OC-01A-21R-A27V-07 |
| TCGA-BS-A0UL-01A-11R-A109-07 |
| TCGA-A5-A0G9-01A-11R-A040-07 |
| TCGA-AP-A05H-01A-11R-A00V-07 |
| TCGA-EY-A2OQ-01A-11R-A19W-07 |
| TCGA-2E-A9G8-01A-11R-A40A-07 |
| TCGA-B5-A11H-01A-11R-A118-07 |
| TCGA-BS-A0UJ-01A-12R-A104-07 |
| TCGA-AJ-A2QN-01A-11R-A18M-07 |
| TCGA-A5-A0GB-01A-11R-A040-07 |
| TCGA-D1-A0ZR-01A-21R-A10J-07 |
| TCGA-D1-A17A-01A-11R-A12I-07 |
| TCGA-AJ-A5DW-01A-11R-A27V-07 |
| TCGA-AP-A05J-01A-11R-A00V-07 |
| TCGA-BS-A0TD-01A-11R-A104-07 |
| TCGA-D1-A2G0-01A-11R-A32Y-07 |
| TCGA-BK-A0CA-01A-21R-A277-07 |
| TCGA-BS-A0TE-01A-11R-A104-07 |
| TCGA-D1-A3DH-01A-11R-A19W-07 |
| TCGA-AP-A0LE-01A-11R-A104-07 |
| TCGA-EO-A3AU-01A-21R-A19W-07 |
| TCGA-A5-A0GJ-01A-11R-A040-07 |
| TCGA-AX-A2H7-01A-12R-A18M-07 |
| TCGA-BG-A0VW-01A-11R-A118-07 |
| TCGA-BG-A0MT-01A-11R-A104-07 |
| TCGA-B5-A0JX-01A-21R-A14M-07 |
| TCGA-FI-A2EW-01A-11R-A17B-07 |
| TCGA-A5-A0GM-01A-11R-A040-07 |
| TCGA-BG-A0MK-01A-51R-A19W-07 |
| TCGA-AX-A06B-01A-11R-A00V-07 |
| TCGA-AX-A1C4-01A-11R-A137-07 |
| TCGA-BG-A0LW-01A-11R-A16W-07 |
| TCGA-AP-A0L8-01A-11R-A040-07 |
| TCGA-BG-A0VZ-01A-11R-A109-07 |
