# Supplementary figures and images for: Development of a senescence-related lncRNA signature in endometrial cancer based on multiple machine learning models
Source: Front Genet. 2025 Nov 27;16:1687922. doi: 10.3389/fgene.2025.1687922 (PMC12694935; doi:10.3389/fgene.2025.1687922)

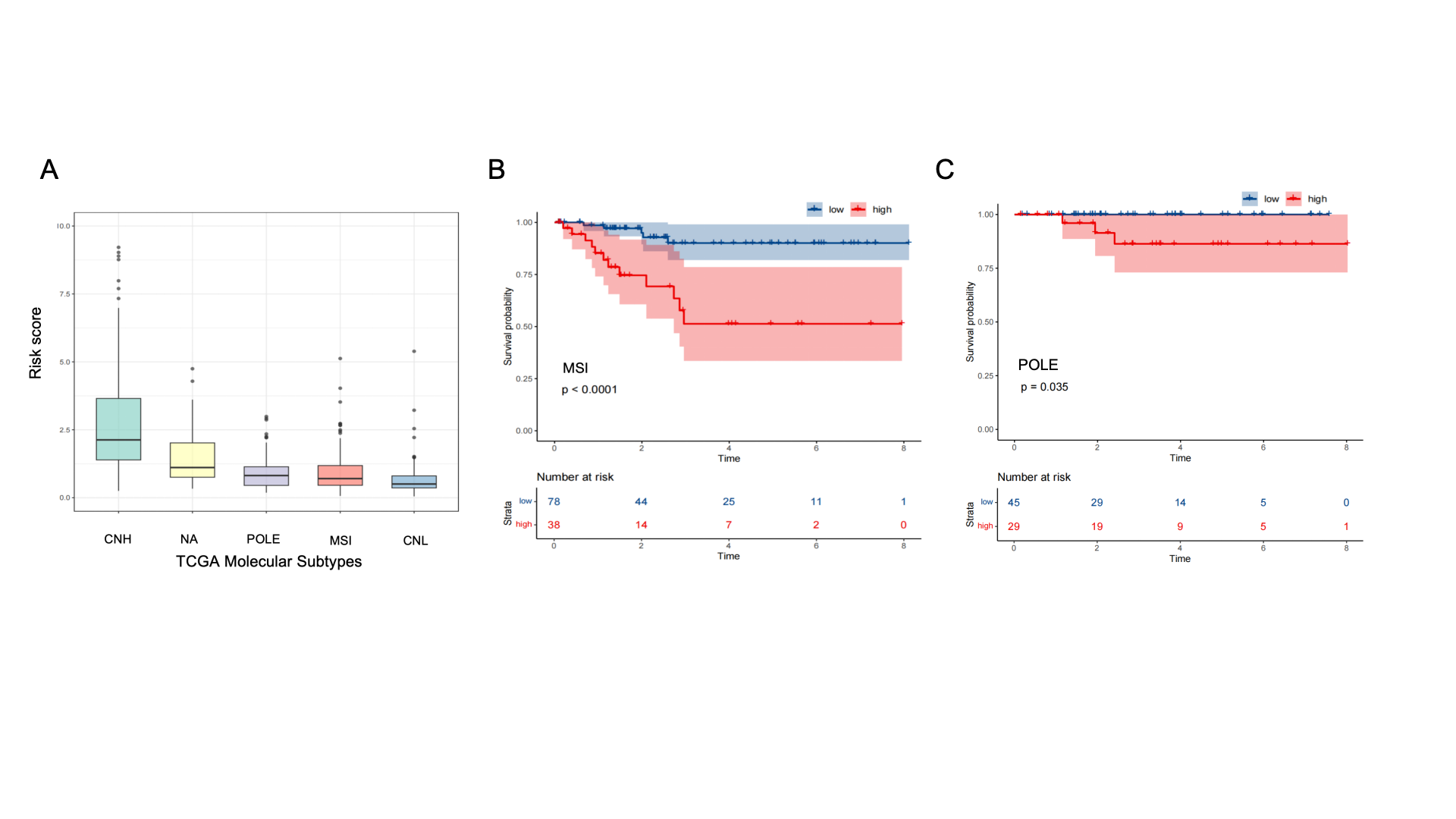

Supplement: Supplementary file 2 [file Image2.tif]

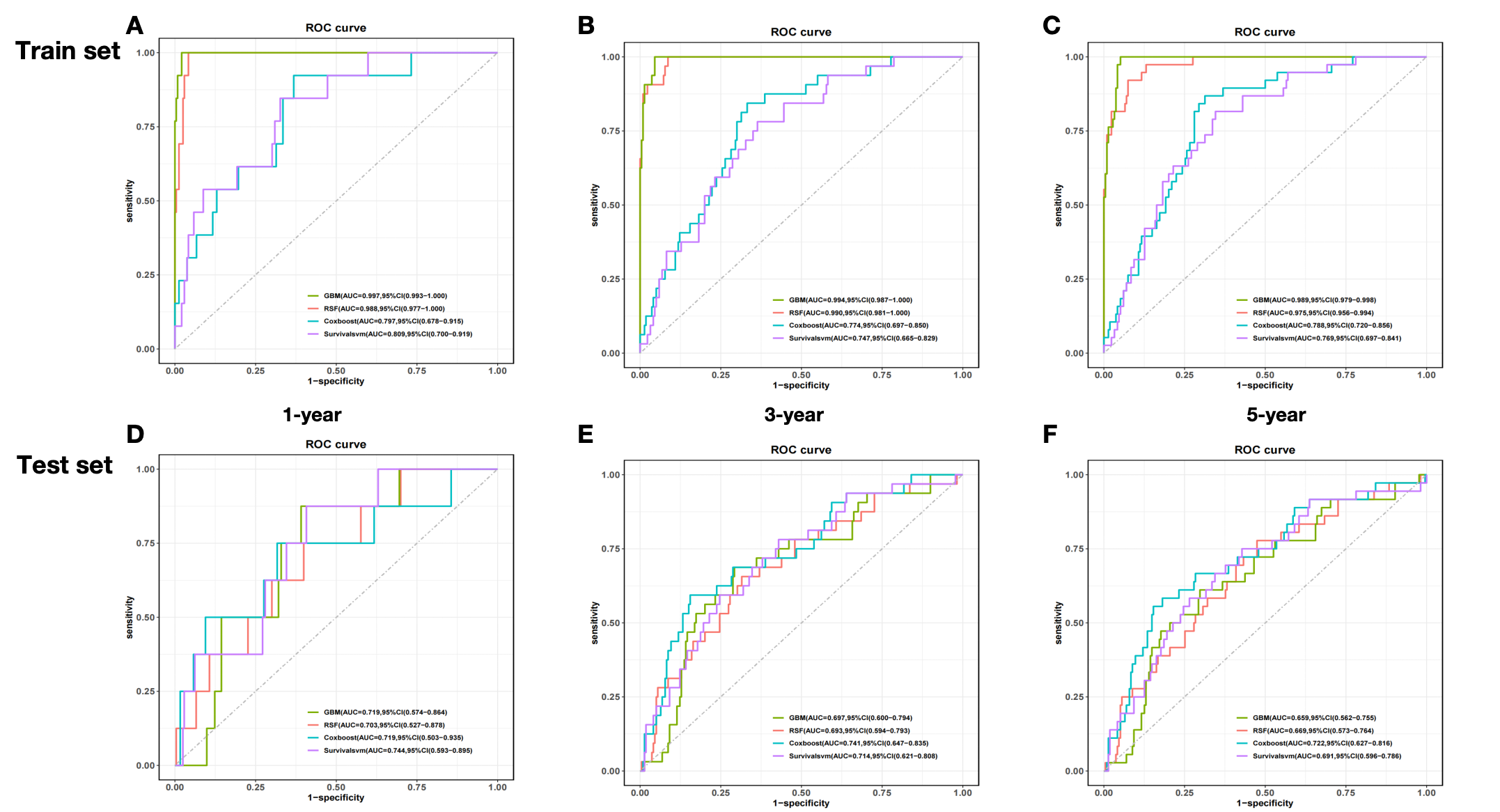

Supplement: Supplementary file 3 [file Image1.tif]
